# GABAergic neurons in basal forebrain exert frequency-specific modulation on auditory cortex and enhance attentional selection of auditory stimuli

**DOI:** 10.1038/s42003-024-07318-8

**Published:** 2025-01-31

**Authors:** Kevin Thomas, Hamid Azimi, Davide Maggioni, Mark Sanders, Pilar Vaca Sánchez, Michael A. Harvey, Gregor Rainer

**Affiliations:** Section of Medicine, University of Fribourg, Fribourg, CH Switzerland

**Keywords:** Sensory processing, Neural circuits

## Abstract

The basal forebrain (BF), in particular its cholinergic projections to cortex, has been implicated in regulation of attention in sensory systems. Here, we examine the role of GABAergic projections of the posterior nucleus basalis (pNB) and globus pallidus (GP) in attentional regulation in the auditory system. We employed a detection task where rats detected a narrow band target embedded in broad band noise, while optogenetically modulating GABAergic BF activity. We found that GABAergic BF modulation impacted target detection specifically close to perceptual threshold, consistent with a role in attentional modulation. We also present evidence for target frequency specificity of this modulation, including frequency selectivity and tonotopic organization of pNB/GP, as well as frequency band specific effects of optogenetics on behavioural target detection and on neural activity in auditory cortex and thalamus. Our findings highlight an important role of BF GABAergic neurons in modulating attention in the auditory pathway.

## Abstract (web summary)

We provide evidence for tonotopy in auditory basal forebrain and document that optogenetic modulation of efferent projections impact neural activity in thalamic and cortical auditory pathway structures, as well as influencing performance in an auditory detection task.

## Introduction

The basal forebrain (BF) contains a set of nuclei, which are important regulators of learning and attention^1^. Investigations have so far focused largely on the cholinergic BF neurons, whose corticopetal axons target wide regions of the neocortex including primary sensory cortices^2^. BF effects on sensory signals are modality specific, such that visual, somatosensory and auditory pathways are regulated by separate sensory-related BF nuclei, namely respectively the horizontal diagonal band (HDB) as well as anterior and posterior nucleus basalis (aNB, pNB)^3–5^. Corticopetal BF projections to primary visual cortex (V1) enhance contrast sensitivity of cortical sensory neurons, as well as modulating tuning and reliability of sensory information encoding^6–12^. These effects are thought to involve mainly projections from the HDB to layer IV, as well as layers II, III of V1. Projections to the primary auditory cortex (A1) also modulate reliability of information encoding, with trial-to-trial response similarity increasing following BF stimulation^13^. Moreover, tone presentation coupled with pNB activation triggers map plasticity, increased neural responsiveness, and enhanced detection of auditory stimuli of the paired frequency^14–17^. These effects appear to be mediated, at least in part, by cholinergic projections to layer I of A1, which are then transmitted to layers II-III and involve a circuit of interneurons with an overall effect of transient disinhibition of pyramidal cells^18^.

The aforementioned cholinergic projections are considered to be a major pathway by which the BF influences cortical activity^19–21^. In addition to the cholinergic neurons, the BF also harbors glutamatergic as well as a population of GABAergic neurons, to which much less attention has been paid, although they make up the majority of BF projections. A better understanding of the BF and its functional role thus requires studies dedicated to other BF cell types. GABAergic BF PV projections trigger striking gamma oscillations in cortical target areas^22^. These BF-PV neurons notably project to cortical interneurons, encompassing both PV or somatostatin positive populations^23,24^. Cortical interneurons play a crucial role in regulating information processing and integration of stimulus-related information in sensory cortices^25^. In the auditory system, interneurons have been shown to modulate various aspects of A1 activity, for example related to spectral surround suppression as well as temporal forward suppression^26,27^. Indeed, direct optogenetic manipulation of A1 cortical interneuron activity has recently been shown to impact auditory task performance^28,29^. Furthermore, PV neurons in the BF also project to subcortical structures, including the thalamic reticular nucleus (TRN), and these projections could thus provide an additional pathway for influencing auditory attention and processing^30^. We thus hypothesize here that BF PV GABAergic projections might contribute to attentional regulation and auditory integration, for example by impacting cortical GABAergic interneuron activation and/or thalamic regulation via the TRN.

While considerable work has linked basal forebrain corticopetal projections to aspects of attentional processing, the specificity of these modulatory influences remains unresolved. Attention can be rapidly shifted, and a corresponding neural mechanism must therefore be able to modulate activity with appropriate resolution and specificity. In terms of temporal specificity, it is becoming increasingly apparent that basal forebrain signalling, at least cholinergic neuromodulation, can act in a phasic manner in the millisecond range, rather than only in the temporally sluggish volume conduction as had previously been thought^31,32^. This high degree of temporal precision is one important prerequisite for attention, which must be rapidly reassigned to pertinent environmental stimuli during natural behaviour. Another important element of selective attention^33,34^, in contrast to more general arousal, is that it must encompass stimulus specificity, allowing attentional mechanisms to select a subset of incoming sensory information based on some attribute, such as orientation or colour in visual processing or tone frequency in auditory processing. While specificity for sensory modality has been documented in the basal forebrain^3^, it is at present unclear whether there is any additional systematic representation of stimulus attributes within each modality. In this study, we address this issue by recording activity in the auditory-projecting pNB/GP nucleus across its anterior-posterior extent, to examine whether a systematic representation of tone frequency exists at the level of single neuron activity across the pNB map. We then examine how optogenetic modulation of specific pNB neuronal populations impacts activity in the auditory pathway using several target frequencies, allowing us to assess specificity of neuromodulatory BF mechanisms.

For a sensitive assessment of auditory performance, we chose auditory task requiring the detection of a narrow-band target sound in the presence of broad-band masking noise. Segregation of behaviorally relevant information from background noise is thought to be an important function of the auditory system involving A1 cortex^35–39^, and is therefore suitable for assessing a potential role of the pNB in auditory attentional selection. In our task, we presented auditory target sounds at various amplitudes, including close to perceptual threshold, which permitted an estimation of target sensitivity in the presence of masking noise. We assess here both up- and downregulation of pNB PV GABAergic neurons, as well as up-regulation of the more general GABAergic population, on auditory pathway activity and auditory detection performance.

## Results

**Fig. 1:**
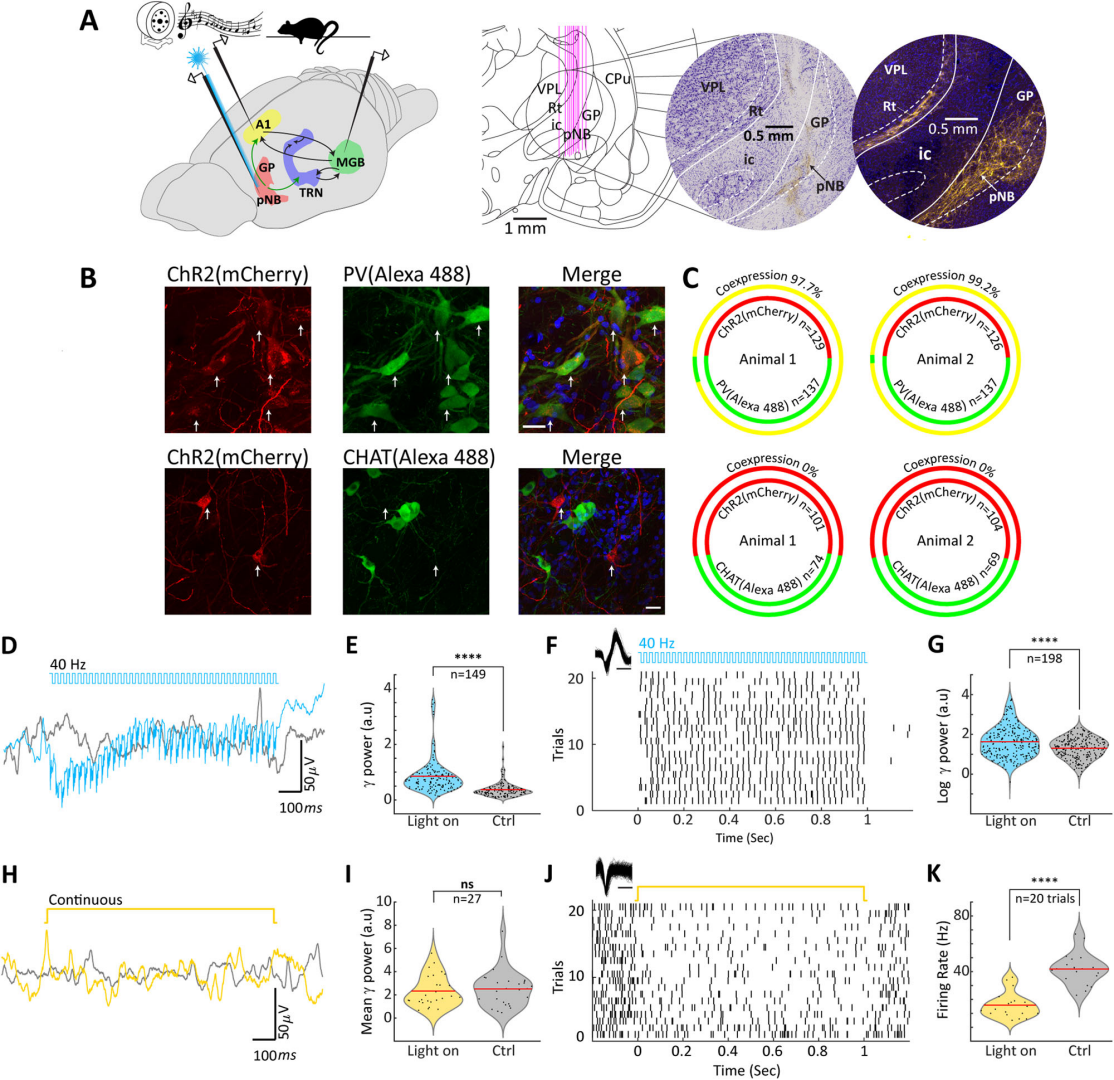
Opsin Expression and Functional Validation of PV-Cre Rats. **A** Left, experimental design for anesthetized recordings, showing the location of optic fiber and recording electrodes in auditory cortex (AC), posterior nucleus basalis / globus pallidus (pNB/GP), and medial geniculate body (MGB). Black and green arrows denote excitatory and PV-inhibitory projections respectively. Right, validation of BF stimulation and recording sites in 12 animals and an example of ChR2-mCherry expression at the injection site. Scale bar 25 μm. **B** Co-localization of ChR2-mCherry with PV-Alexa 488 (top) and CHAT-Alexa 488 (bottom). **C** Top, quantification of co-expression, yellow ring, for ChR2-mCherry, red, and PV-Alexa 488, green, *N* = 2 animals. Bottom, same as top but for CHAT-Alexa 488, green, and ChR2-mcherry, red, *N* = 2. **D** Mean LFP response to 40 Hz, 473 nm blue light ChR2 stimulation of pNB/GP PV neurons, blue, as compared to control (light off), grey, in a single animal. Note that during laser stimulation LFP activity is locked to the light frequency. **E** Violin plot of gamma power (30–80 Hz) of 149 LFP recording sites in 12 animals during pNB/GP PV Chr2 stimulation, and control, red line indicates the mean. **F** Example raster plot showing the spiking activity of a putative PV neuron over 20 trials of 40 Hz ChR2 stimulation. Inset shows the units’ wave forms, scale bar 0.5 ms. **G** Violin plot of gamma power (30–80 Hz) for the population of 198 pNB neurons during Chr2 PV light stimulation (blue) and control (gray), red line indicates mean value. **H**–**J** Same as (**D**–**F**), but with 594 nm yellow laser Arch inhibition of pNB/GP PV neurons. **K** Violin plot of firing rate for an individual pNB/GP neuron that was significantly modulated during Arch mediated optical inhibition (yellow) and control (gray), red line represents the mean.

We first conducted histological and electrophysiological studies to characterize neural responses to auditory stimulation in brain regions of interest. We performed simultaneous recordings in the basal forebrain (BF), targeting posterior nucleus basalis and the surrounding region of globus pallidus (pNB/GP), the auditory medial geniculate body (MGB) of the thalamus, and the auditory cortex (AC) under isoflurane anaesthesia (Fig. 1A, left panel). Prior to these recordings, rats were bilaterally

injected with appropriate viral constructs (PV-ChR2, PV-Arch, mDlx-ChR2) in the pNB/GP, in order to selectively manipulate local BF GABAergic circuits. Targeting of the pNB/GP was verified using Nissl staining as well as opsin expression (Fig. 1A, right panels and Supp. Materials 3). We verified co-expression of both ChR2 and Arch with PV neurons in the pNB/GP (Fig. 1B), with ChR2 expression restricted to PV-positive GABAergic and not ChAT-positive cholinergic pNB neurons in two rats (Fig. 1C). We employed mDlx to target inhibitory BF populations, and document robust co-localization with PV as well as GAD67 populations. We found less than 2% colocalization with ChAT neurons and no colocalization with somatostatin positive cells (Supplementary Material4). In electrophysiogical validation studies, we recorded local field potential (LFP) and spiking activity in pNB/GP to provide evidence for functional opsin expression. For ChR2, we observed locking of the LFP to laser stimulation delivered at 40 Hz, as shown for an example recording in Fig. 1D. Quantifying this using the evoked mean gamma power in the frequency band 30–80 Hz, we observed reliable entrainment of gamma oscillations in pNB across recording sites (*n* = 149 recordings, *n* = 12 animals, see Fig. 1E). Similarly, reliable spiking activity was triggered by 40 Hz laser stimulation, as shown by the example unit in Fig. 1F, as well as by the entrained spiking at gamma frequency observed across the population of recorded pNB neurons (*n* = 198 neurons, n = 12 animals, see Fig. 1G). For Arch, as we used continuous laser illumination for inhibiting neural PV circuits there was no observable effect on gamma band LFP activity (see Fig. 1H, I), likely due to a floor effect as BF circuits generally exhibit reduced activation during anesthesia. We did however observe significant reduction of responses in the population of neurons that were significantly modulated by laser illumination (paired t-test comparing period of light illumination to pre-illumination baseline activity, *p* < 0.05, total recorded neurons *n* = 35, *n* = 2 animals, Fig. 1J–K).

**Fig. 2:**
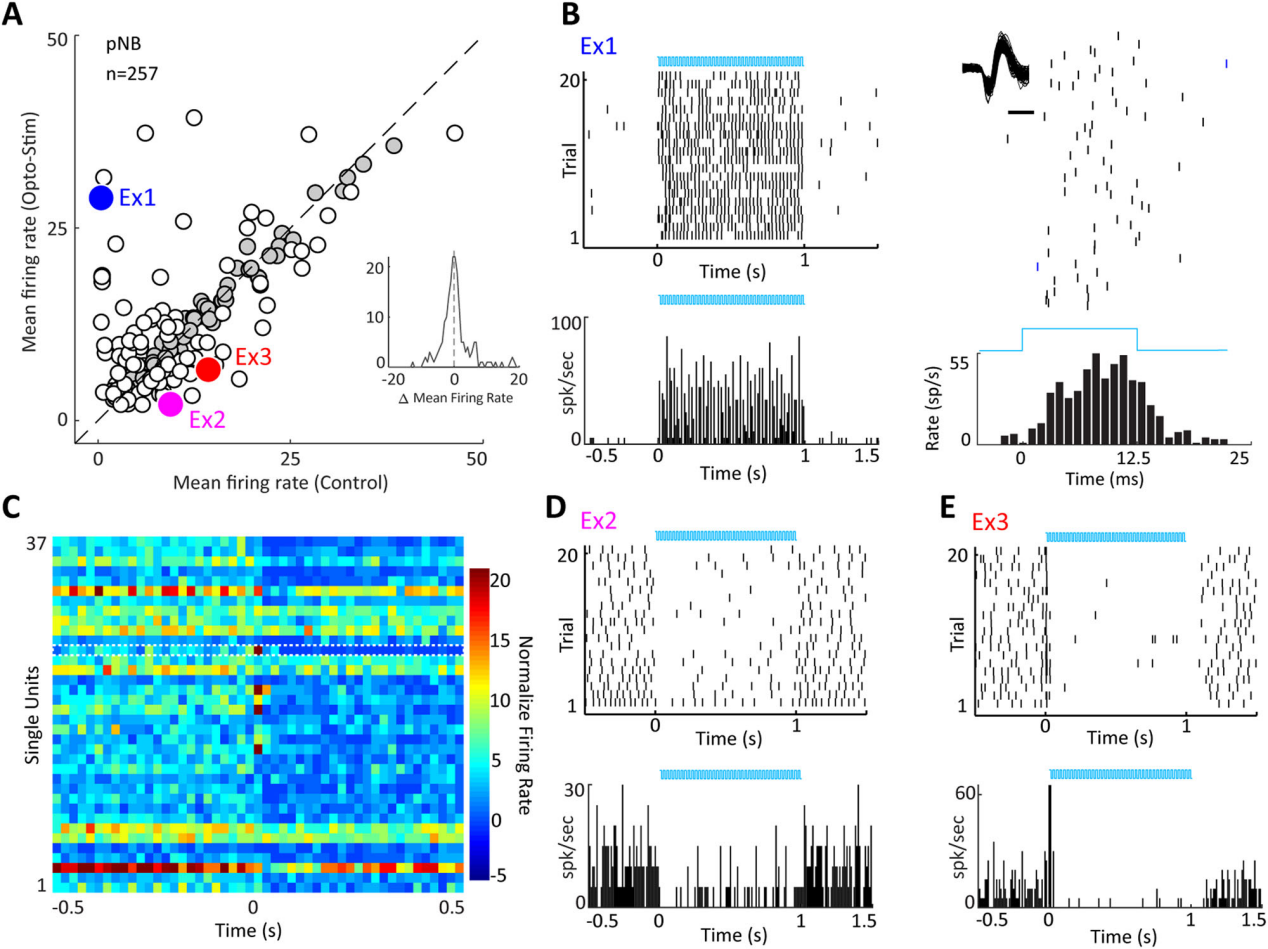
Effects of pNB PV ChR2 stimulation on neurons in the pNB. **A** Mean firing rate in the laser off vs laser on conditions for all cells. White circles represent neurons showing significant modulation (up or down) by laser stimulation, gray circles represent cells not responding to laser stimulation. Large blue, pink and red circles are the example neurons shown in (**B**, **D**, **E**) respectively. **B** Top Left, raster plot of a neuron excited by optical stimulation, every vertical tick represents a spike, and sequential rows represent trials. Bottom Left; peristimulus time histograms (PSTH) bin size, 5 ms, showing the mean firing rate during laser stimulation. Top Right; Raster plot and PSTH (bin size, 1 ms) for laser-evoked spikes aligned to rising edge of each laser pulse. Wave forms shown in inset, scale bar 500 μs. **C** Heat map for the population of the neurons in pNB inhibited by laser stimulation (*n* = 37), color bar normalized firing rate. Each square represents the mean firing in 20 ms bins before and after laser onset. Dashed white line outlies the activity of the example neuron shown in (**E**). **D** Raster plot and PSTH for a pNB neuron inhibited by laser stimulation. **E** Raster plot and PSTH for a neuron that shows low latency excitation followed by long lasting inhibition during laser stimulation.

Activation of PV neurons resulted not only in excitatory, but also in inhibitory responses within pNB/GP. Indeed, a similar number of neurons were excited (*n* = 42) and inhibited (*n* = 37) by 40 Hz laser ChR2 activation of PV neurons, paired t-tests on the activity of individual neurons comparing light illumination with pre-illumination baseline, *p* < 0.05, *n* = 257 neurons, *n* = 12 rats, Fig. 2A). The excited population typically exhibited elevated spiking activity triggered by laser pulses at short latency, as seen in an example neuron in Fig. 2B and as expected for the targeted PV GABAergic pNB/GP neurons. For 38 units (*N* = 7 rats) that showed a significantly enhanced response to light stimulation (paired t-test comparing spiking during light stimulation to baseline at *p* < 0.01), the mean latency to the first action potential following light stimulation onset was 5 ± 2 ms. Plotting the activity profile (Fig. 2C) of the neurons inhibited by ChR2 activation (paired t-test on mean firing rate during baseline period vs. ChR2 period for individual neurons, *p* < 0.05), we noticed a subset of neurons (n = 5/37, 14%) where inhibition followed a brief initial transient excitation lasting 20–40 ms after laser pulse train onset, while most inhibited neurons showed no such transient excitatory response, (*n* = 32/37, 86%). Example raster plots, shown in Fig. 2D, E, illustrate the response profile of these two distinct populations. The occurrence of inhibition in the pNB/GP following ChR2 activation of PV GABAergic neurons is suggestive of recurrent circuit activations triggered by laser pulses and likely involving other BF cell types. Purely inhibited neurons are likely non-PV, whose activity is suppressed by local GABA release following PV neuronal activation, whereas the transiently activated cells might themselves be PV neurons that receive delayed inhibition through local circuits or other mechanisms. Note that BF PV neurons have previously been thought to not target local cholinergic and somatostatin-positive GABAergic cells, and only weakly target the glutamatergic population^21^. Nevertheless, our results suggest that ChR2 activation of PV neurons has at least three distinct effects on the local neuronal circuit probably involving distinct cell types.

**Fig. 3:**
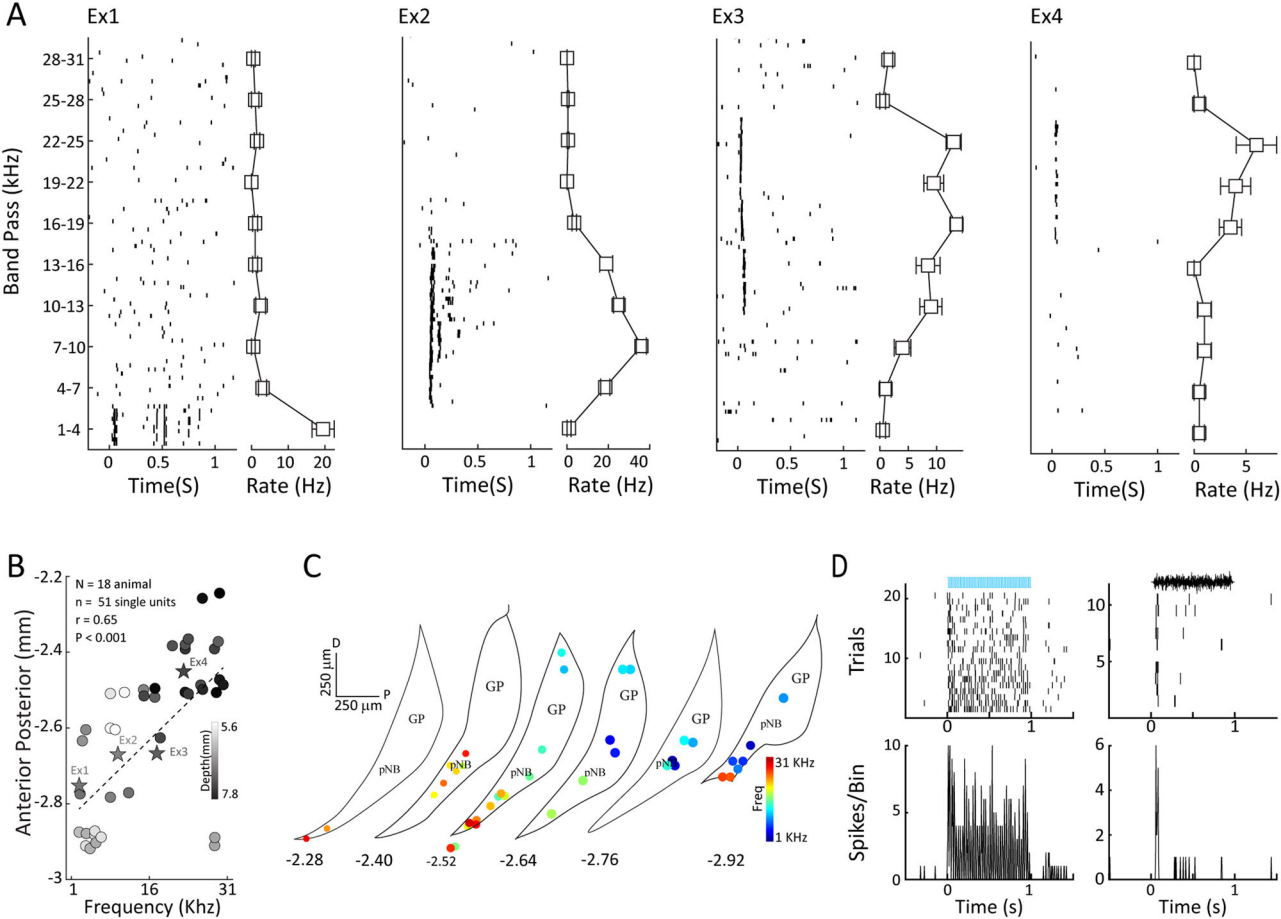
Tonotopic organization in pNB. **A** Raster plots and mean firing rates for four frequency tuned single units in the pNB during the presentation of ten different band-passed noise stimuli. Error bars SEM. **B** Anterior-posterior location in the pNB is plotted against preferred frequency for each unit. The color gradient from white (Dorsal) to black (Ventral) represents the depth of the recorded single unit in pNB. Stars labeled as (Ex1 to Ex4) represent example units shown in (**A**). **C** Anatomic location of units shown in (**B**). Color gradient from blue (low frequency) to red (high frequency), represent the units’ best frequency, GP globus pallidus. **D** Rasters and PSTH’s for an optotagged, putative PV neuron, showing both short latency excitatory responses to laser stimulation in the absence of auditory input, left, as well as low latency auditory responses, right. This is the same unit as Ex2, and also exhibits frequency tuning.

To assess frequency tuning in pNB/GP in the PV-CRE rats, we recorded neural activity to various band-pass noise frequency stimuli in the audible range of the rat. A considerable number of pNB/GP neurons were frequency-tuned, and four examples are shown in Fig. 3A, with preferred frequencies ranging between 1 and 25 kHz. When examining the anatomical distribution of these neurons along the extent of the pNB/GP, we found evidence for tonotopy in that preferred frequency was significantly correlated with both anterior-posterior and dorsal-ventral recording position (AP: *r* = 0.65, *p* < 0.001, DV: *p* < 0.001, *n* = 51 single neurons see Fig. 3B). The reconstructed anatomical recording positions are shown in Fig. 3C, demonstrating a gradual progression from low to high preferred frequency of neurons recorded within the pNB/GP. This finding, which has in fact been previously observed in guinea pig^40^, is also generally consistent with the documented functional topography for the cholinergic basal forebrain^41^. It has previously been shown that ChAT positive neurons in the pNB that project to AC exhibit low latency frequency tuned responses to auditory stimulation^15^. In that study, Guo and colleagues^15^ had in fact also observed auditory responses in a non-cholinergic population of the caudal basal forebrain, although it was hitherto unclear whether these were GABAergic neurons. In order to assess if the PV neurons in our study were also responsive to auditory stimuli, we employed an opto-tagging strategy. We found 29 single units that demonstrated low latency responses to ChR2 laser stimulation, and deemed them to be putative PV neurons. Of these, 6 cells showed both low latency ( < 50 ms) response to auditory stimulation as well as significant frequency tuning, Fig. 3D, indicating that PV neurons are among the frequency-selective neural population in the pNB/GP.

**Fig. 4:**
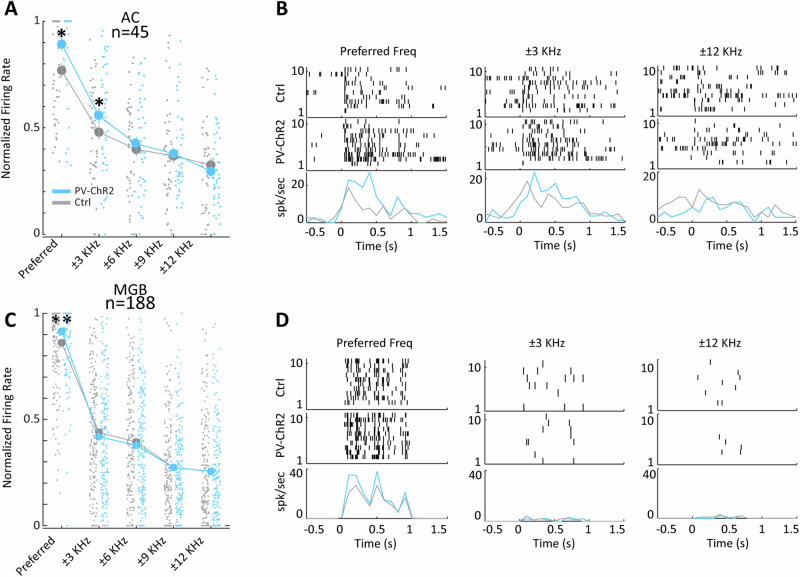
Frequency specific AC and MGB modulation by BF PV-ChR2 stimulation. **A** Normalized firing rates at preferred frequency bands and four adjacent bands for all AC units showing frequency tuning, small grey and blue dots represents individual data points for the control and opto conditions respectively. Error bars SEM. **B** Raster plots and PSTHs for 3 example cortical neurons showing enhanced firing following BF PV-ChR2 stimulation at their preferred and adjacent frequency band (left and center), but not at more distant bands (right). Auditory stimulus onset at T = 0 s. **C**, **D** Same as (**A**, **B**) but for single units recorded in MGB, note that as opposed to AC significant effects were not found for adjacent frequency bands in the MGB.

The evidence for tonotopic organization of pNB is of substantial interest, as it opens up the possibility of frequency-specific influence of basal forebrain modulatory circuits on auditory processing, as indeed Chernychev and Weinberger had speculated previously^40^. Thus, we proceeded to record the activity during optogenetic activation of pNB/GP PV GABAergic neurons. Within the pNB/GP, we observed general enhancement of neural activity (Supplementary Material5). We proceeded to identify 188 and 45 neurons in MGB and AC respectively (*N* = 18 rats, both areas studies simultaneously) that were frequency tuned (one-way ANOVA on firing rates to each band-pass stimulus in both control and optogenetics conditions, df = 19, *p* < 0.01). We then identified the preferred frequency for each neuron, as the band-pass frequency where maximum firing rate occurred in either of the two conditions ensuring an unbiased estimate, and computed normalized rates for the preferred and adjacent frequency bands. We found that ChR2 PV pNB stimulation enhanced firing rate at the preferred and ±3 kHz adjacent frequency in AC (repeated measures ANOVA, Bonferroni-corrected post-hoc tests, *p* < 0.05), while in MGB only the enhancement at the preferred frequency was significant (see Fig. 4A, C). The single neuron examples for AC and MGB (see Fig. 4B, D) highlight the specific enhancement of neural activity in the auditory pathway at the preferred frequency. These results are compatible with a frequency-specific, overall excitatory modulatory influence of pNB on auditory pathway activity, as the PV GABAergic projection neurons, by a disinhibitory influence, are expected to trigger enhanced prinicipal cell activations in AC. Another possibility is that local modulation of BF PV circuits impacts close-by cholinergic corticopetal projection neurons which might contribute to these observed results. In addition, indirect effects on the auditory thalamus mediated through the TRN could also play a role, a hypothesis supported by the robust presence of BF PV axons innervating the TRN (Supplementary Material 6). Interestingly, we found that optogenetic activation of BF PV neurons had small to negligible effects on spontaneous activity in both regions, with only 4 of 55 MGB units (7%, paired t-test *p* < 0.05) and 10 of 189 A1 units (5%, paired t-test *p* < 0.05) showing significant effects. The BF modulations occurred thus specifically during auditory stimulation and did not impact baseline firing in absence of sound stimulation, at least from the data from the anesthetized preparation analysed here.

**Fig. 5:**
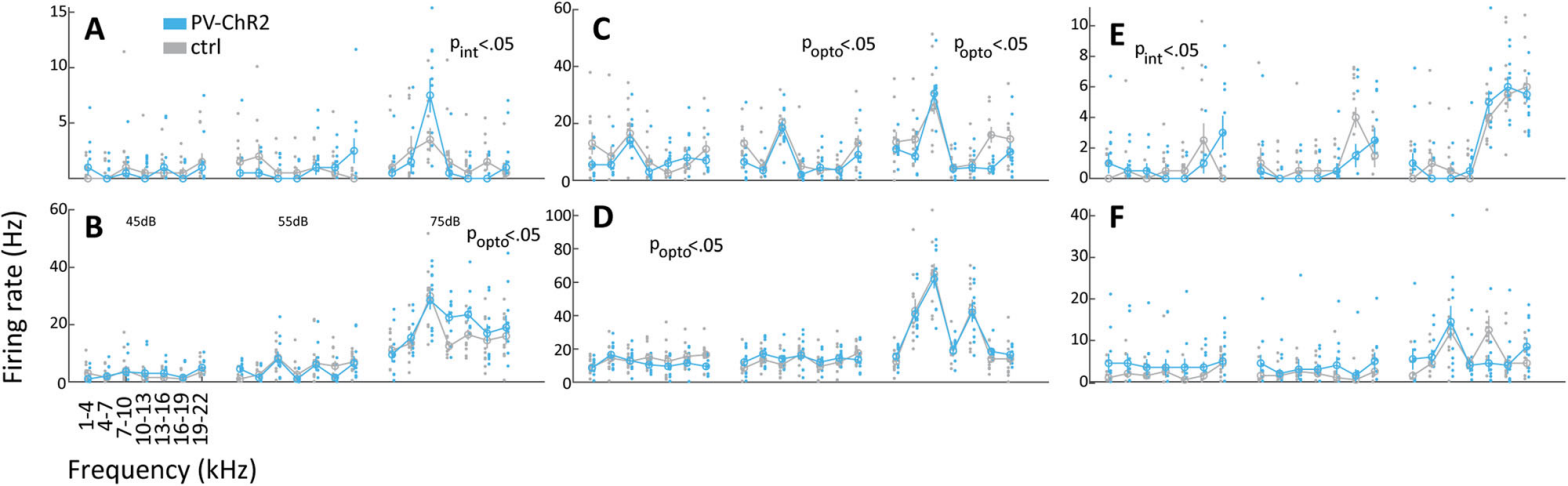
Example neurons illustrating the variety of effects of BF PV ChR2 stimulation on MGB responses in the presence of broad band noise. **A**–**F** Each panel consists of three subpanels, showing activity of a single MGB neuron at low, medium and high SPL. The *P*-values mentioned denote a significant effect of optogenetic activation or a significant interaction between frequency tuning and optogenetic activation at the corresponding target SPL. Small grey and blue dots represent individual data points for the control and opto conditions respectively. Error bars SEM.

As our task involved the detection of target sounds in the presence of noise, we performed additional neural recordings in MGB and AC using presentations of brief segments of narrow-band target sounds at three different sound amplitudes in the presence (mask condition) and absence of broadband masking noise (control condition). The broadband mask generally suppressed responses to narrow band target sounds at the preferred frequency in both regions, although the effects were variable from cell to cell (Supplementary Material 7). We performed additional experiments to examine neural responses to targets in the presence of the mask during BF PV-ChR2 activation compared to control conditions. In the MGB, we obtained data from 117 neurons in 4 animals. We performed separate ANOVAs at each of the three target sound amplitudes, with factors of sound frequency and PV-ChR2 stimulation. We found that 35/117 (30%) of MGB neurons exhibited either a main effect of light or an interaction frequency/light (2-way ANOVAs, *p* < 0.05), demonstrating that optogentic BF PV activation modulated activity in the MGB. Considering the global effect on firing rate across all frequency bands, we observed an overall enhancement of activity in 19 neurons and a reduction in 15 neurons. Responses of six example MGB neurons are shown in Fig. 5, illustrating a variety of activity modulations: we observed activity enhancement at the preferred frequency and at adjacent frequencies (Fig. 5A, B), generally compatible with the notion that BF PV ChR2 might make the target sound more detectable, although these effects were seen at high amplitude in these examples. Another type of modulation can be described as suppression of responses at non-preferred frequencies (Fig. 5C, D), which could also aid detection of target sounds. Finally, other neurons exhibited more complex modulations, such as a combination of enhancement and suppression (Fig. 5E) or more broad activity enhancement particularly at low and medium sound amplitudes (Fig. 5F). Irrespective of the exact type of modulation, our recordings highlight that BF PV-ChR2 activation robustly modulates MGB activity, providing a functional pathway by which the basal forebrain could impact behavioral detection of target sounds.

**Fig. 6:**
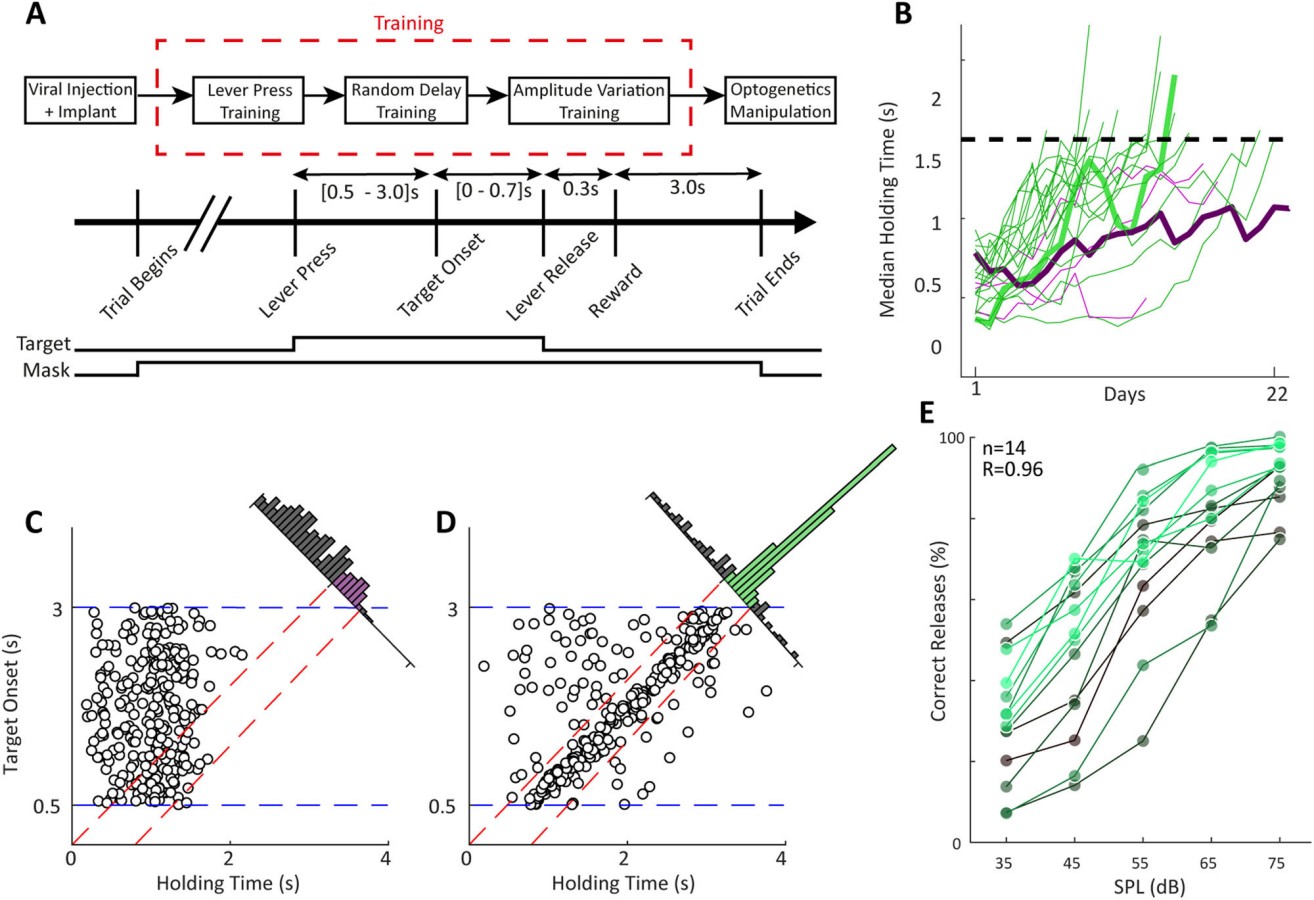
Behavioral training design. **A** Behavioral experimental protocol (top), and schematic of a typical trial. **B** Acquisition of auditory target detection task at high target amplitude, as quantified by median lever holding time during successive training days. Green lines represent animals that achieved performance criterion; purple lines represent animals failed to reach criterion. **C** Example lever hold time plot for an animal that failed to acquire the task corresponding to thick purple line in (**B**); lever releases were unrelated to target onset and tended to cluster below 1.5 s. **D** As in (**C**), but for an animal that successfully learned the task (thick green line in **B**), such lever releases were restricted to the response window. **E** Correct release percentage varied systematically with target sound amplitude after six days of training. Individual rats are represented by different color shades.

To examine the behavioural impact of optogenetic modulation of BF GABAergic circuits, we employed an auditory detection task that allows for an assessment of auditory sensitivity (Fig. 6A). Rats were trained to hold down a lever and release it when they perceive a target sound (bandpass noise 4–7 kHz or 7–10 kHz) in the presence of a broadband mask (1–22 kHz). We used two target sounds to allow examination of frequency-specificity of pNB modulatory effects on behavior. We used uniform background masking auditory noise and different target sound amplitudes to facilitate the study of auditory perception close to threshold, guided by our accompanying electrophysiological studies (see above). After viral injection and optic fibre implantation (see methods), rats were initially trained to hold a lever. Initially, a high amplitude target sound was presented upon lever press, and animals were automatically given a sugar pellet upon lever release. In the next training phase, a random length delay was introduced between the lever press and the target sound presentation, and this delay was successively incremented up to a total duration of 500 ms + 1–2500 ms (randomized). Animals had 700 ms from target sound onset to respond by releasing the lever upon target detection. The number of training days required to achieve criterion performance of 1.5 s median holding time varied between 6 and 21 days depending on individuals (see Fig. 6B). Some animals disengaged from the task before achieving criterion performance and were excluded from further study. Response times of an animal that failed to learn are shown in Fig. 6C for an example session. The lever release times appear unrelated to the target sound onset, but instead release times were linked simply to the lever hold duration, yielding a near uniform release time histogram (Fig. 6C, inset). By contrast, for a rat that successfully acquired the task, release times were clearly linked to the target sound onset (Fig. 6D), confirmed by the high density of responses in the 700 ms time window following target sound presentation (Fig. 6D, inset). Subsequently, lower amplitude target sounds were introduced alongside the high amplitude sounds, and after 6 training days with multiple amplitudes, training was considered complete. At this point, rats for which correct release percentage, i.e. ${hits}/\left({hits}+{misses}\right)$, was significantly correlated with target sound amplitude (*r*-values range: 0.94–0.99, *p* < 0.05, see Fig. 6E) were selected for optogenetic manipulations.

**Fig. 7:**
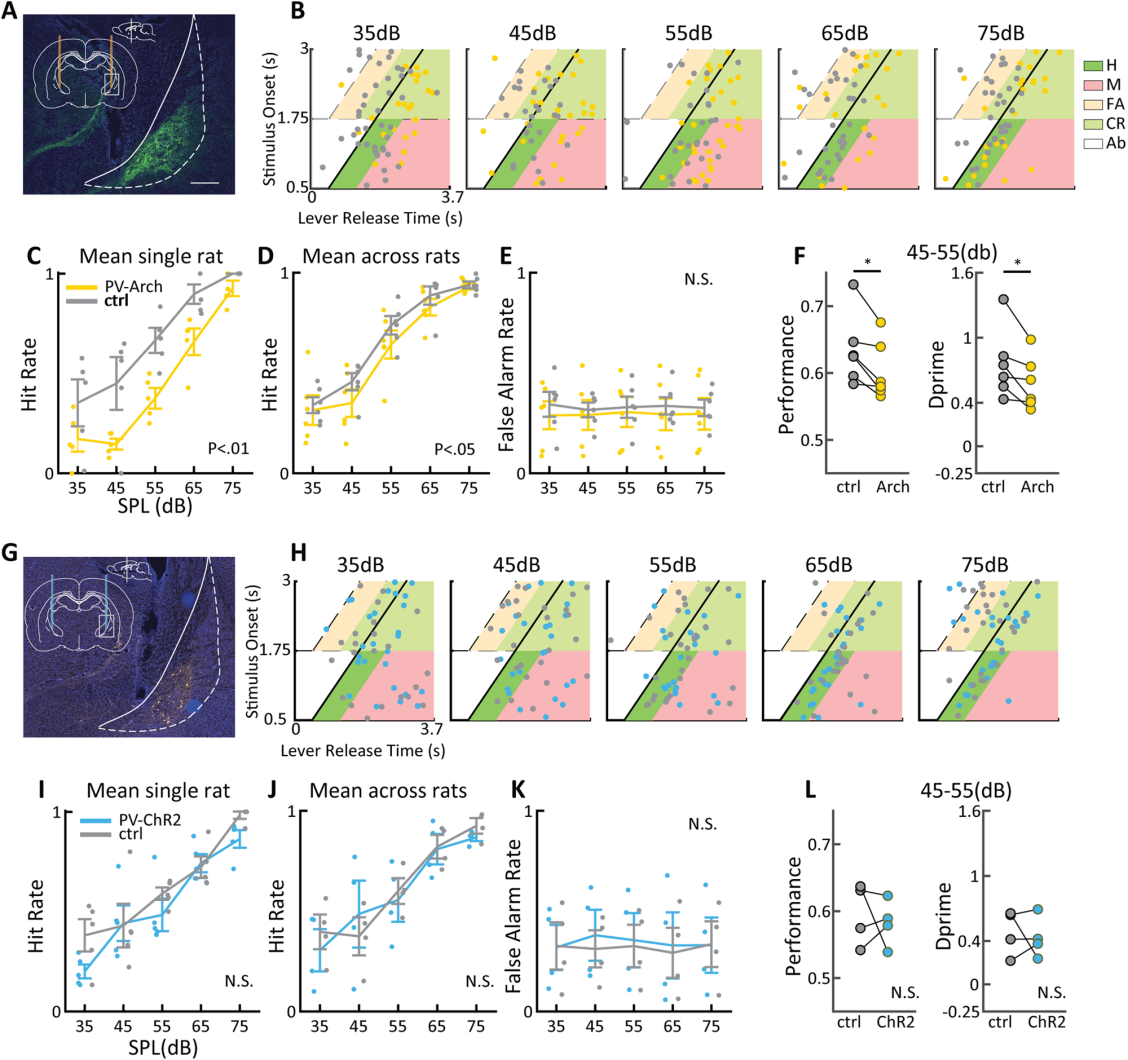
Behavioral effects of BF PV-Arch inhibition and ChR2 activation with 7-10khz targets. **A** Arch-EYFP expression in pNB in an example animal. Scale bar 0.5 mm. **B** Lever release times for an example session at five different SPLs. Target onset is indicated by the thick, oblique line. The colored trapezoids refer to hit (H), miss (M), false alarm (FA) and correct rejection (CR) and abort (AB) responses (see legend). **C** Mean hit rate across all behavioral sessions (*n* = 5) for the animal in (**B**) in the light stimulation vs. control condition, small dots indicate individual data points. **D**, **E** mean hit rate and false alarm rate across animals (*n* = 6) using aggregate data from each rat. **F** Mean performance and d’ for the same data using SPL near perceptual threshold. Error bars reflect SEM. * denotes *p* < 0.05. **G**, **L** Same as (**A**, **F**) above but for BF PV ChR2 (n = 4 animals).

Trials with optogenetic modulation and control trials were randomly interleaved during each behavioural session. We first examined the impact of Arch silencing of PV neurons on target sound detection. Post-mortem histological analysis confirmed specificity of Arch expression within the pNB/GP, as illustrated in the example shown in Fig. 7A. We used a signal detection framework to estimate various behavioral parameters including hit rate, false alarm rate and behavioral sensitivity (d’) comparing optogenetic activation to control conditions by dividing behavioral responses into four categories relative to target sound onset (see methods). Example behavioural results for the different amplitudes in a single behavioral session are displayed in Fig. 7B, illustrating that for high amplitude targets (75 dB), rats made few “miss” responses in both conditions, suggesting that the rat had no problem detecting the target. At lower target amplitudes, “miss” responses became more prevalent, and this appeared to be more pronounced during inhibition of BF PV neurons. Taking into account all behavioral sessions from this animal (*n* = 5), we confirmed that the hit rate (${hits}/\left(\right.{hits}+{misses}$) was significantly reduced during light stimulation (2-way ANOVA with factors light stimulation and SPL: main effects of SPL and light stimulation *p* < 0.01, Fig. 7C). Indeed, a reduced hit rate during light stimulation was also significant across the population of PV-Arch group animals (*N* = 6, 2-way ANOVA: *P* < 0.05, Fig. 7D). In our framework, errors can arise from a reduced hit rate or an increased false alarm rate. As light stimulation did not impact false alarm rate significantly (2-way ANOVA, *P* > 0.1, Fig. 7E), effects were therefore specific to auditory stimulus related behavior, compatible with the lack of baseline activity modulations in MGB and AC in the absence of auditory stimuli presented above. To further characterize behavioral impact of PV Arch stimulation, we focused on behavioral performance averaged across 45 dB and 55 dB sound amplitudes, which were close to perceptual threshold, observing significant reductions in both behavioral performance and d’ sensitivity (see methods, Fig. 7E, F). Our analyses verify that inhibition of BF PV neurons decreased behavioral performance for 7-10 kHz targets as measured by multiple parameters related to auditory target detection. On the other hand, the same was not true for 4–7 kHz targets, where inhibition of BF PV neurons had no effect on target detection performance (Supplementary Fig. 8). This difference is unlikely to result from differences in peripheral sensitivity between 4–7 and 7–10 kHz targets, as there was no difference in hit rate between these two target frequencies in the control condition (2-way ANOVA with factors SPL and target frequency, *P* > 0.1 for effect of target frequency and interaction). Further, these results were independent of the target frequency used in initial training, which was counterbalanced (*N* = 3: 4–7 kHz and *N* = 3: 7–10 kHz). Taken together, Arch silencing of BF PV neurons impacted auditory detection task performance, causing an attentional deficit in target detection at low to intermediate SPLs, consistent with an impact on sensory perception. In addition, we present behavioral evidence for frequency specificity of BF PV influence on auditory perception, consistent with tonotopic organization of pNB/GP described above.

We next analysed effects of ChR2 activation of BF PV neurons on auditory detection performance, after verifying correct localization of ChR2 in pNB (see Fig. 7G). Behavioural task performance for an example rat for target sound 7–10 kHz is shown in Fig. 7H, I, using the same procedure described above. The analysis demonstrates that activation of the BF PV population had no significant effects on hit rate, nor did it modulate false alarm rate, performance or d’. These results were consistent across animals (Fig.  7J–N). For target sound frequency 4–7 kHz, we also found no significant modulations in detection performance of rats (Supplementary Material 9). We conclude that BF PV ChR2 activation had no noticeable effects on auditory task performance, despite the fact that auditory pathway activity modulations were observed in our anaesthetized recordings (see Figs. 4, 5). We suggest that this apparent discrepancy is due to the effect of anesthesia on BF activations. As pNB PV neurons may already be adequately activated during awake state task performance, further increase in their activation does not appear to be beneficial or detrimental within the parameters tested in our study.

**Fig. 8:**
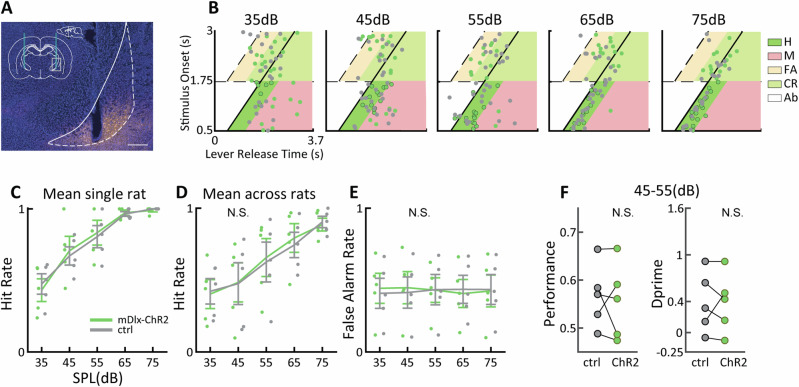
Behavioral effects of BF mDlx ChR2 activation with 7–10 khz targets. **A** mDlx-EYFP expression in pNB/GP in an example animal. Scale bar 0.5 mm. **B** Lever release times for an example session at five different SPLs. Target onset is indicated by the thick, oblique line. The colored trapezoids refer to hit (H), miss (M), false alarm (FA) and correct rejection (CR) and abort (AB) responses (see legend). **C** Mean hit rate across all behavioral sessions (*n* = 5) for the animal in (**B**) in the light stimulation vs. control condition. **D**, **E** mean hit rate and false alarm rate across animals (*n* = 5) using aggregate data from each rat. **F** Mean performance and d’ for the same data using SPL near perceptual threshold. Error bars reflect SEM. Small dots indicate individual data points in (**C**– **E**).

As the activation of PV neurons by itself had little effect on behaviour, we wondered whether more general activation of GABAergic BF circuits might have a more pronounced impact. We employed mDlx-ChR2 for this purpose, which permits activation of PV but also other GABAergic cell types, but excluding the somatostatin population within the BF (Supplementary Material 4). Note that the higher coexpression of mDlx-ChR2 with GAD67 compared to PV suggests that other inhibitory BF cell types are activated in the mDlx-ChR2 condition. As above, we first verified probe locations and expression within the region of interest. We found that, similar to PV-ChR2, mDlx-ChR2 stimulation had no significant effect on any of the aforementioned behavioural parameters for the 7–10 kHz (Fig. 8B–F) or 4–7 kHz (Supplementary Material 10) targets. Overall, neither the specific activation of BF PV neurons, nor the activation of a more general population of BF GABAergic neurons had any effect on auditory target detection. As there were no systematic differences in LED probe placement between behavioral groups (see Supplementary Material 11), our behavioral findings are likely due to specific modulation of pNB/GP neuronal cell types, and do not result from any biases in LED probe placement.

## Discussion

We have shown that Arch-silencing of BF PV neurons impairs the detection of auditory target sounds in the presence of background masking noise. As BF PV neurons specifically target cortical interneurons^23,24^, Arch inhibition of BF PV projection neurons would be expected to result in disinhibition of cortical interneurons with a subsequent increase in activity in their target cells. In this sense our findings are consistent with recent reports that direct cortical ChR2 activation of PV A1 interneurons suppresses auditory responses and impairs auditory detection performance in the mouse^28,29,42,43^. However, the effects of modulating A1 interneurons directly and indirectly via the pNB/GP were not identical: while Lakunina and colleagues observed that stimulation of interneurons directly caused an amplitude-independent performance deficit^29^, Arch inhibition of the pNB/GP in our study affected detection of target sounds at amplitiudes close to perceptual threshold. The latter effect is more akin to an attentional function, as attentional effort is required to filter a weak target from noise while strong salient targets are readily detected with minimal effort. We suggest that the BF PV projections to AC may target specific subsets of cortical interneurons that support attentional filtering, and that direct optogenetic manipulation of AC interneurons may recruit additional populations leading to less specific effects^43^. However, potential direct targeting of cortical interneurons by BF PV projections is only one of several potential pathways that might underlie the effects observed here. Additional experimental studies, for example silencing cholinergic pathways during BF PV activation or light stimulation of BF PV axon terminals in AC, would be needed to provide more definitive evidence. As it stands, two other pathways might provide the neural substrate for the electrophysiological and behavioral effects observed here. For example, BF PV projections to the MGB via the thalamic reticular nucleus (TRN) might also contribute to the effects, as this pathway also acts in a disinhibitory fashion on auditory processing. The presence of PV+ fibers in the TRN supports this hypothesis, as does functional evidence that BF PV-ChR2 activation modulates MGB activity to target sounds in the presence and absence of a broadband mask. It is also possible that the effects reported in this study might be mediated indirectly through cholinergic corticopetal projections, which are modulated by local synaptic processing within the BF downstream of PV light stimulation. Indeed, our evidence regarding multiple distinct effects of BF PV ChR2 activation on local circuit activity is consistent with an indirect activation of cholinergic projections. Our neural recordings also show that broadband noise degrades auditory stimulus evoked neural activity more in MGB than AC, in line with previous findings^28^, suggesting that cortex appears able to extract useful behaviorally relevant auditory information from degraded sensory thalamocortical inputs. Reduced AC tone sensitivity in the presence of additive auditory noise has been observed in other studies^44,45^, and has been linked to increased detection thresholds for auditory targets across mammalian species including humans^46,47^.

We found that ChR2 activation of pNB PV neurons had no effect on auditory target detection, which is likely due to the fact that BF PV neurons already tend to be activated during wakefulness, consistent with reports that activation of these neurons promotes wakefulness and cortical activation^19,21^. Based on the expected disinhibitory effect on AC pyramidal cells described above, ChR2 pNB/GP PV activation would be expected to reduce AC interneuron activity. Direct optogenetic suppression of A1 interneuron activation however has been shown to both decrease the amplitude of responses to tones^42,43^ and to impair detection of auditory targets in background noise^29^, suggesting that the pNB/GP inhibitory projection targets only a subpopulation of A1 interneurons. Our findings thus show that adequate pNB GABAergic activation appears necessary for proper attentional filtering in the auditory system, and that the contributions of the BF PV circuits play a significant role that is likely coordinated with the more established BF cholinergic projection circuits. In addition to activating specifically the pNB/GP PV cell population, we also examined the effect of more global activation of pNB/GP GABAergic neurons, targeting a more general population of GABAergic BF neurons using the mDlx enhancer. This manipulation, similar to PV-ChR2 activation, also failed to produce any behavioral effects in our task. As our colocalization analysis shows, the majority of the labelled GABAergic BF neurons were in fact PV neurons, such that the functional equivalence of PV-ChR2 and mDlx-ChR2 is unsurprising from this perspective. We found no evidence of somatostatin (SOM) positive cells in the BF region of interest, although these were found laterally near the GP border and outside the region of viral expression. Therefore, BF SOM+ neurons exhibited little colocalization with viral expression and this GABAergic population was thus not directly modulated by light stimulation in our study. It has been shown that SOM neurons exhibit no prominent long-range projections, but profoundly inhibit local circuit activity^21,48^. Furthermore, experimental work in the ventral pallidum has implicated SOM neurons in gamma oscillation regulation and behavioral state regulation^49,50^.

It is useful to contrast these findings to the impact of activation PV neurons in other nuclei of the BF. As described above, pNB/GP PV neuron activation enhances activity in the auditory pathway and triggers an attentional focused state that facilitates detection of low amplitude target sounds. Activating PV neurons in the ventral pallidum (VP) and magnocellular preoptic nucleus (MCPO) on the other hand tends to trigger an internally focused, self-directed brain state where attention is withdrawn from external sensory stimuli^49,51–53^. Based on these results and other evidence from human imaging data^54,55^, several nuclei of the BF are now considered to be important subcortical nodes of the default mode network (DMN). The DMN is complementary to the dorsal attention network in humans^56^, and these two networks are active in alternation and govern overall brain state. Our findings suggest that BF PV neurons can play a role in enhancing both DMN- and attentional brain functions, depending on the BF nucleus. The present findings thus reinforce the notion that the BF is not a functionally homogenous region but supports a diversity of functions. Not only the BF cell type, but also the particular BF nucleus needs to be considered for a comprehensive understanding of BF contributions to cortical state regulation.

A key novel aspect of our results is the frequency specificity of BF modulatory effects on auditory pathway activations and auditory stimulus based behaviour. We have five lines of evidence in support of frequency-specificity of pNB/GP PV neuromodulatory impact on auditory processing. (1) We have evidence for frequency tuning in BF PV neurons, obtained using optogenetic tagging and demonstrating short latency excitatory responses to ChR2 activation as well as frequency selectivity. While frequency tuning had been observed previously in the pNB and other BF nuclei, specifically also in cholinergic neurons^15,40^, our study is the first to confirm frequency tuning in the pNB/GP PV population. (2) We present evidence for tonotopic organization of the pNB/GP, such that preferred auditory frequency was correlated with anterior-posterior as well as dorsal-ventral axes. While tonotopy has been previously observed in the nucleus basalis of the barn owl^57^, our study shows that systematic representation of sound frequency is also present in the rat BF and this may generalize to other mammalian species. Frequency tuning, in conjunction with tonotopy, provides a scaffolding for potential attentional regulation of auditory processing. Indeed, we found frequency specific effects of BF modulation at electrophysiological as well as behavioral levels in our study. (3) PV-ChR2 activation of pNB/GP enhanced responses to target stimuli in absence of the broadband mask in AC and MGB at the preferred, and for AC also the immediately adjacent frequency band, while having no effect on distant frequency bands. This provides a potential mechanism for how BF PV neurons might accentuate task-relevant sensory information at a particular frequency of interest, rather than causing global non-specific increases in auditory pathway activation. (4) We found particularly in the MGB evidence that BF PV-ChR2 activation modulated the activity of frequency tuned neurons in the presence of the broadband mask, in a manner that might enhance the neural representations of the target sounds in the auditory pathway. (5) Our behavioral findings also lend support to the idea of frequency specific BF PV modulation, in that the PV-Arch behavioral effects were restricted to a specific frequency band and did not occur for an adjacent control frequency band. Taken together, our evidence suggests that BF PV neurons exert a substantial impact on auditory pathway activations and auditory task performance, and are crucial particularly for detecting targets where correct performance requires focused attention. We suggest that the BF may be an important component for regulation of auditory attention, which is indeed thought to exert frequency-specific effects^58,59^. We suggest that the pNB/GP PV projections are likely coordinated with pNB cholinergic projections^15^ by PV neuron activation. It has already been shown that cholinergic neuromodulation, unlike noradrenergic neuromodulation, is modality specific and auditory, somatosensory and visual pathways can be individually modulated by cholinergic projections, whereas noradrenaline provides a more general, diffuse influence across sensory systems^3,60,61^. The present study suggests that, in addition, the neuromodulatory effects can also exhibit specificity within a modality, i.e., by priorizing a certain frequency band that happens to be of behavioral significance. It is highly probable that the GABAergic projections are activated in concert with cholinergic input to cortex. Further work is needed to reveal how these BF modulatory influences are coordinated across cell types and BF nuclei to contribute to attentional functions and behavioral state regulation.

## Material & Methods

### Subjects

A total of 40 Long-Evans PV:Cre rats of both sexes, aged between 90 and 180 days old, were used in this study. Rats were maintained on a 12/12 light/dark cycle with food and water available ad libitum until the beginning of the experiment. The local ethical committee on animal experimentation (canton of Fribourg) approved all experimental procedures. We have complied with all relevant ethical regulations for animal use.

### Anesthetized experiments

#### Surgery

14 animals of both sexes were used for this procedure. Anesthesia was induced with ketamine (100 mg/kg) and xylazine (20 mg/kg) and maintained with inhaled isoflurane ≈1.0% in pure oxygen. Animals were placed in a stereotactic frame, and a midline incision was made on the scalp, the skin and periosteum were reflected, and the temporal muscle was retracted in order to expose the skull overlying auditory cortex. Small craniotomies were made for recordings from posterior nucleus basalis, (pNB), anterior/posterior (AP) − 2.3 Medial/Lateral (ML) 3.8) and ventral medial geniculate body, MGB, (AP − 7.8 and ML 3.4). We targeted the ventral MGB for these recordings (see Supplementary Material 1). A larger craniotomy was made exposing most of the temporal lobe for AC recordings. Post mortem recording site reconstructions showed that the majority of our AC penetrations were centered on A1, we cannot exclude the possibility that some recordings were from secondary auditory areas (see Supplementary Material 2). All recordings were made with tungsten microelectrodes (FHC, Bowdoin, USA) with impedance ≈450 kΩ. The MGN electrode was advanced under electrophysiological guidance, using a hydraulic micromanipulator, until short latency auditory responses were observed. The A1 electrode was then inserted into the auditory cortex, using the medial cerebral artery as a location marker for consistency throughout the animals. Finally, a tungsten microelectrode coupled with a 100 µm diameter optic fiber was lowered to about 1 mm above the target location in pNB. After each recording session, electrodes were lowered, to record activity of different cells. Body temperature was maintained with a homeostatic heating blanket (Kent Scientific) at 37 ^◦^C throughout the whole procedure. We studied neural activity in 12 animals using PV-ChR2 and 2 animals using PV-Arch^62^.

#### Auditory stimulation and electrophysiology

Binaural auditory stimuli were delivered in an open field configuration through an electromagnetic speaker (TDT) placed 10 cm in front of the animal’s nose. The speaker was controlled with an RZ6 auditory processor (TDT). We used band pass noise: Seven 1 s segments of band pass noise stimuli were constructed by filtering (4-pole Butterworth) random white noise with a constant band width of 3 kHz (1–4, 4–7, … to 22 kHz). Each of these segments was either presented at with a Low, Medium or High amplitude, corresponding to 45, 55 and 75 dB SPL. Finally, an auditory masking noise, consisting in a broad band pass noise (1–22 kHz) was played in half of the trials at 60 dB. Each recording block contained four presentations of each of the stimulus condition: one coupled with optogenetics stimulation and masking noise, one with sole optogenetics stimulation, one coupled with masking noise only, and one control condition. In total 10 or 20 repetitions were presented for the control and stimulation conditions. Intertrial intervals were randomized at 0.5 ± 0.2 s. An optic fiber of diameter 100 µm was attached to a microelectrode and connected to a 473 nm blue laser (Changchun New Industries Optoelectronics, China). Laser intensity was set to 3.5 mW using an optical power meter (PM 100D, Thorlabs Newton NJ). Stimulation was delivered using 40 Hz square pulses with a duty cycle of 50%. The stimulation protocol was controlled by a pulse generator (Rigol, Beaverton OR) triggered by a TTL from the RZ5 recording device. Signals for single-unit analysis were acquired through a unity gain head stage (TDT) and digitized at 24 kHz, and band pass filtered between 300 Hz and 8 kHz using an RZ5 amplifier (TDT) and stored on a PC for offline analysis. Subsequent spike sorting was performed offline using Offline Sorter software (Plexon). LFP data were sampled at 2.4 kHz, and band pass filtered between 0.5 and 300 Hz.

### Behavioral experiments

#### Surgery

26 rats of either sex were used for the behavioral experiments. The anesthetic regimen and midline incision were the same as for the anesthetized recordings described above. Additionally, the animals received ophthalmic ointment to prevent desiccation of the eye. Two small burr holes were made for 1 µl bilateral injections of of viral construct, either AAV5-EF1a-DIO-eArch3-EYFP (*n* = 9), AAV5-EF1a-DIO-hChR2(H134R)-mCherry (*n* = 6) or AAV5-mDLX-Chr2-mCherry (*n* = 10). The viral infuser was left in place for 10 minutes following each injection before it was slowly removed. Immediately after viral injection, bilateral pNB 240 µm optic fiber (Doric lenses) were implanted using the same coordinates as for viral injection, except for 4 rats in the PV-Arch group where we employed a wireless LED stimulation system (Neurolux, Chicago, USA) and the appropriate bilateral device was implanted instead of the optic fibers. Stability of the implant was ensured with screws in the skull and cemented into place with dental acrylic. Animals were subjected to food scheduling and maintained at a minimum of 90% of their normal weight.

#### Behavioral task and training

Behavioral sessions were conducted in an operant chamber (32x32x55cm) equipped with a lever, a house light, and a reward light at the feeding through. A camera and a speaker were mounted 50 cm above the arena. The operant chamber was controlled by a PC using custom MATLAB scripts. The auditory stimuli used for behavioral experiments were constructed in the same way as for the electrophysiological experiment. Animals were initially trained and tested using a 4–7 kHz target followed by testing on a 7–10 kHz target with the exception of the additional animals in the PV-Arch group (*n* = 4), for which the target frequency order was reversed.

In Phase 1, animals were first manually shaped to press a lever in order to receive a food reward (45 mg chocoloate flavored pellets, TestDiet Richmond, USA). During initial training, each lever press immediately triggered the presentation of the target stimulus (300 ms duration). In Phase 2, animals were trained to wait until target presentation before releasing the lever. The same auditory target as in phase 1 was played after a delay following lever press, and the animal had to respond within a time window to receive a food reward. Responding before the target was presented or after the response window, resulted was followed by a 10 s time-out. The delay between lever press and target presentation was gradually increased, and the response window decreased, step by step throughout phase 2, see supplementary table 1. After rats accomplished step 6, they were moved to phase 3, where the target was presented at five different sound pressure levels (35, 45, 55, 65, 75 dB) for at least six training days. For quantitive analyses in PV-Arch and PV-ChR2 groups, we included animals that showed a hit rate of 0.80 or greater at 75 dB and a hit rate of 0.60 at 35 dB or less averaged across light-on and control conditions. As animals in the mDlx-ChR2 group were sighltly less well trained, we used criteria of 0.75 and 0.65 cutoff values.

For each trial, lever pressing had a 50% chance of triggering optic manipulation, which lasted as long as the lever was pressed down. Behavioral data were gathered using custom MATLAB scripts. Routine behavioral and electrophysiological analysis were performed using custom routines written in MATLAB. During task performance, rats were always rewarded for responding within 700 ms of the target sound. To facilitate the use of a signal detection theory, we divided the trial time into four parts using a previously published method^63^. “Hits” were defined as lever releases within the 700 ms response window corresponding to target onset times from 0.5 s to 1.75 s, while lever releases after the response window were defined as “misses”. False alarms were defined as releases in a time window identical to the hit rate window but in absence of the presentation of a target, i.e. stimulus onset 1.75 s to 3 s. Lever releases following the “false alarm” window were assigned as “correct rejections”. From these parameters, we computed performance ($\frac{{HR}+{CR}}{{HR}+{CR}+{FA}+{MS}}$), HR: number hits, CR: number correct rejections, FA: number false alarms and MS: number misses as well as behavioral sensitivity (d’). Lever releases occurring prior to the hit and false alarm windows were considered as aborted trials and not used for further analyses. Lever releases occurring before target onset were randomly assigned to an SPL level, and estimates for false alarm rate, performance and d’ are based on an average for 100 random assignments.

#### Optogenetics

For tethered stimulation, a 200 μm dual branching patch cord was connected to the external portion of the chronically implantable optical fibers (Doric Lenses, M3-thread). Patch cords were attached to a 473 nm blue laser or to a 594 nm yellow laser (Changchun New Industries Optoelectronics, China) for ChR2 and Arch rats respectively. Laser intensity was set to 3.5 mW using an optical power meter (PM 100D, Thorlabs Newton NJ). This value was chosen based on previous experience in our laboratory and the results of the anesthetized electrophysiology. In ChR2 animals, stimulation was delivered using 40 Hz square pulses with a duty cycle of 50% (Rigol, Beaverton, USA) triggered by a TTL from the RZ5 recording device. For wireless optogenetics, we used a 590 nm yellow LED at setting of 10 mW; a value also chosen based on previous validation studies.

#### Histology

Rats were deeply anesthetized with 100 mg/Kg pentobarbital, then perfused transcardially with 400 mL 0.1 M phosphate bufferred Saline (PBS) (pH 7.4) followed by 400 mL cold 4% paraformaldehyde in 0.1 M PBS. Brains, then were placed in the same fixative overnight at 4 °C. Whole brains where then rinsed 3 times for 20 minutes each, in cold 0.1 M PBS and cryoprotected by immersion in sucrose gradient (15 and 30% w/v sucrose) until they sank. Brains were then blocked and fast frozen in dry‐ice chilled isopentane and stored at least overnight at −20 °C before cryosectioning. Brains were cut into 40 μm‐thick,coronal sections using a sliding microtome (MICROM HM 440E, Microm International GmbH, Walldorf, Germany). Every third section was kept in a storage solution (30% ethylene glycol and 30% glycerol in 0.1 M Phosphate Buffer) at −20 °C. To determine electrode track positions, sections were mounted on Super Frost ® Plus Adhesion slides(Fisher Scientific AG, Reinach CH),stained for nuclei and Nissl bodies, using a 0.5% Cresyl violet (acetate) and coverslipped using EuKitt ® Quick‐hardening mounting medium (Sigma‐Aldrich Chemie GmbH, Switzerland). To visualize the virus‐mediated expression of ChR2‐mCherry and Arch‐eYFP by fluorescence, sections where mounted, air‐dried, and cover slipped using an aqueous Vectashield ® antifade mounting media with Dapi (Vector Laboratories, Inc., Burlingare, CA, USA, H‐1200). Nissl Stain and virus expression were imaged using a NanoZoomer 2.0‐HT slide scanner (Hamamatsu Photonics), with a 2 × 20 0.75 NA air objective and at a resolution of 0.23 µm/pixel. Visualization and analysis was done with the NDP.view 2 freeware (Hamamatsu Photonics). Immunohistochemistry: We performed immunohistochemistry for the specific neuronal markers ChAT and PV on adjacent sections from the same brain specimens extending anterior to posterior through the pBF (coordinates from Bregma ‐1.80 mm to Bregma ‐2.52) spaced every sixth 40‐μm thick section for each marker at regular intervals. Free‐floating sections were prepared by rinsing in 0.1 M phosphate buffered saline (PBS) for 20 min, then sections were treated with Heat Induced Epitope Retrieval (HIER) in a Tris‐EDTA based solution at pH 9.0 to unmask the antigens and epitopes and thus enhance staining intensity of antibodies. Then sections were permeabilized for 90 min with 0.1 M PBS + 0.3% Triton X‐100, followed by incubation in a blocking solution containing 0.1 M PBS‐ 0.05% Triton X‐10, 0.3 M Glycine, and 10% Normal Donkey Serum (NDS) (Abcam, AB 7475) for 2 hours at RT. Thereafter, sections were incubated with mouse anti‐PV (1:5000; Swant, Switzerland, 235Pur) and goat anti‐ChAT (1:200; Millipore, AB144P) in 0.05% Tween 20, 5% NDS and 0.02% NaN3 in 0.1 M PBS for 48 h at 4 °C. Sections were washed in 0.1 M PBS + 0.05% Tween 20 + 1% NDS four times, 20 min each, and then incubated in Alexa Fluor‐488‐tagged donkey anti‐mouse IgG (1:400; Jackson Immuno Research, Europe Ltd, UK; Cat N.: 715‐545‐151) or Alexa Fluor‐488‐ tagged donkey anti‐goat IgG (1:200; Sigma‐Aldrich Co., St Louis, MO, USA, Cat N.: SAB4600032), or Alexa Fluor‐594‐ tagged donkey anti‐mouse IgG (1:800; Jackson Immuno Research, Europe Ltd, UK; Cat N.: 715‐585‐150) for 16 h at 4 °C. Sections were then counterstained with DAPI at 0.5 μg/mL for 5 min. and mounted on Super Frost Plus microscopic slides, and cover‐slipped with 90% glycerol + 0.5% N‐propyl gallate in 20 mM Tris pH 8.0. Images were collected using a 1.30 NA 63X glycerol‐immersion objective on a Leica TCS SP5 Laser scanning confocal microscope (Leica Microsystems AG, Switzerland)) and analyzed using LAS X software (version 3.3.0, Leica Microsystems). Image processing was performed with the ImageJ/Fiji software (NIH, Bethesda, MD, USA). Quantification using stereological methodology: To estimate the number ChR2‐mCherry and Arch-eYFP positive cells that co‐labeled with ChAT positive neurons and PV positive neurons in the pBF, we employed the unbiased stereological technique using the optical fractionator workflow in MBF Stereo Investigator (Version 2019.1.3, MicroBrightField, Williston, VT, USA), in combination with a Zeiss Axioplan Fluorescence microscope (Zeiss, Jena, Germany), connected to an Orca‐05 G monochrome digital Camera (Hamamatsu, Japan) and coupled with a motorized x‐y precision stage system BioPoint 2 (Ludl Electronic Products, LTD, NY, USA). Cells were counted with a 63x NA 1.40 oil immersion objective with a mean section thickness of 32.4 μm. The pBF was sample in every sixth consecutive 40 μm section, yielding 8 for analysis (PV (*n* = 4) or ChAT (*n* = 4) respectively. The pBF region of interest was first identified under 5x magnification. A 70 × 70 μm counting frame was used and a sampling grid area (xy) that covered the region of interest. At each sampling location, the microscope was focused down through the dissector sample to count any cell within that particular counting frame. Only clear cell bodies were counted. Guard zones of 0.5μm at the top and bottom of each slice were used with an optical dissector height of 25 μm. The pBF region of interest extended from −1.80 mm anterior to −2.52 mm posterior to bregma. Two ChR2 were analyzed. Counts represent the mean from 4 four sections per animal.

### Statistical analyses reproducibility

Experiment details are provided in the text and “Methods”. All the statistical analyses were performed inMatlab. Biological replicates are denoted with a upper case N, and technical replicateds with a lower case n. Technical replicates refer to repeated measures and biological replicates refer to separate animals.

### Reporting summary

Further information on research design is available in the Nature Portfolio Reporting Summary linked to this article.

## Supplementary information

Supplemental Material

Reporting summary

## Data Availability

All data utilized in this study are freely available for download at 10.5061/dryad.0zpc8675w.
