# Supplementary material for: GABAergic neurons in basal forebrain exert frequency-specific modulation on auditory cortex and enhance attentional selection of auditory stimuli
Source: Commun Biol. 2025 Jan 31;8:149. doi: 10.1038/s42003-024-07318-8 (PMC11785998; doi:10.1038/s42003-024-07318-8)
Supplement: Supplementary file 1 — Supplemental Material [file 42003_2024_7318_MOESM1_ESM.pdf]

Supplementary Material 1: Projected Trajectory of Medial Geniculate Body Recordings

Projected Trajectory of Medial Geniculate Recordings

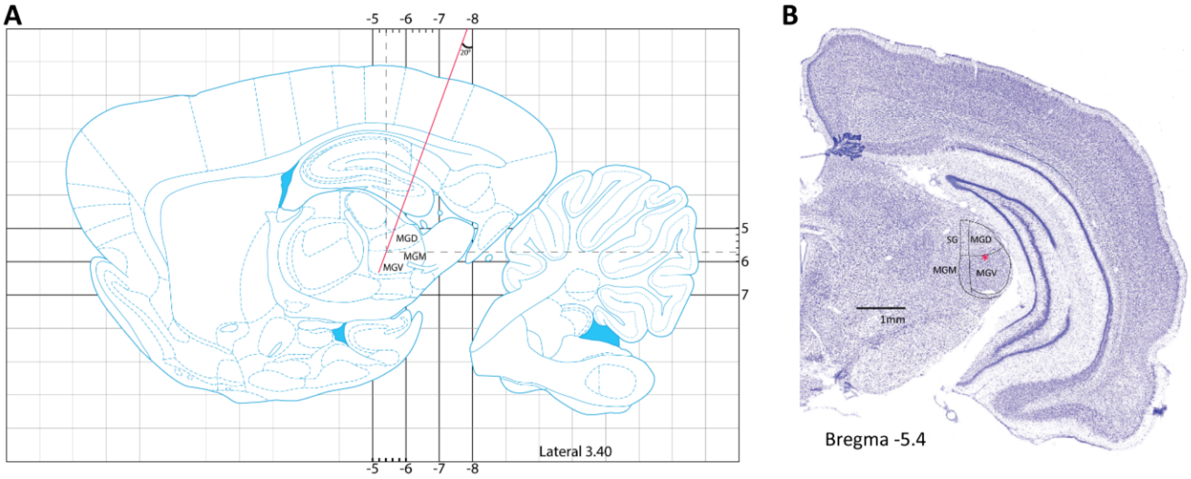

(A) Sagittal diagram of the rat brain from Paxinos and Watson showing the planned trajectory of the electrode penetration (red line) to the ventral area of the MGB (MGBv). Electrodes were inserted at a 20° angle AP -7.8 and ML 3.4 from bregma. Dashed lines indicate the anterior-posterior and dorsal-ventral locations at the entry into MGBv. (B) Nissl stained frontal section at the AP coordinates of entry into the MGBv. Red star reflects the planned location of the electrode tip at these coordinates. Note that at these coordinates the MGBm is relatively small compared to the MGBv.MGD, medial geniculate dorsal, MGV medial geniculate ventral, MGM, medial geniculate medial, SG, suprageniculate.

We stereotactically targeted ventral MGB (vMGB) for the thalamic recordings, as shown in the schematic above. We therefore consider that a majority of our thalamic recordings are from this region, but cannot exclude a contribution of neighbouring mMGBm, although this region is substantially smaller in volume at the anterior-posterior target locations.

Supplementary Material 2: Localization of Recording Sites in Auditory Cortex

Localization of Recording Sites in Auditory Cortex

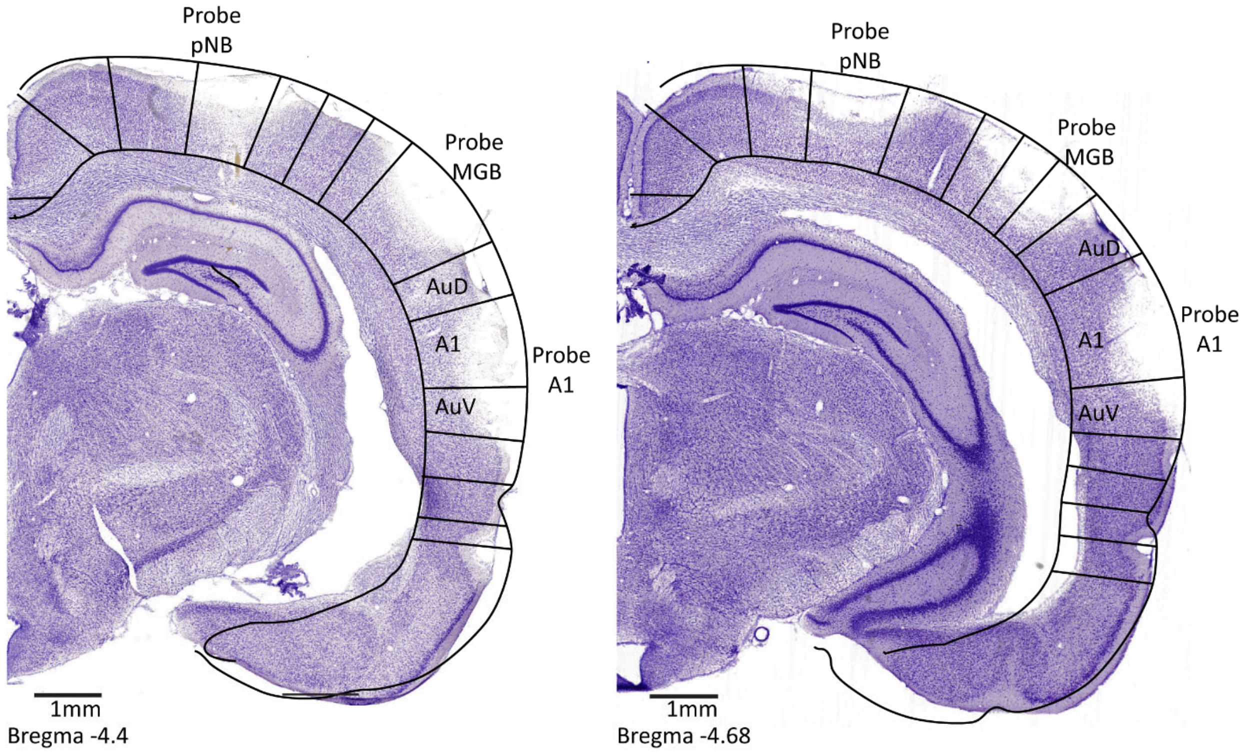

Nissl stained sections from two animals show the cortical areas recorded from. Note that the outline of the craniotomies for our three probes, pNB, MGB, and A1, are clearly visible, and A1 recordings were primarily targeted to the center of the craniotomies labeled Probe A1. The micrographs reveal that while recordings were targeted to anatomically defined primary auditory cortex, there is a possible inclusion of secondary auditory cortical areas, particularly the ventral aspect. AuD, dorsal secondary auditory cortex, A1, primary auditory cortex, AuV, ventral secondary auditory cotrex.

In order to localize our cortical recording sites, we refer to the lesion marks created as a result of our craniotomies and multiple electrode penetrations. This reveals that while a preponderance of our penetrations targeted anatomically defined A1 (*Rat Brain Stereotaxic Coord.*, 1982), we cannot rule out the inclusion of some neurons from secondary areas, most notably the ventral aspect of secondary auditory cortex. In a recent study (Chavez and Zaborszky, 2017), it has been shown that the projections of non-cholinergic, presumably GABAergic, pNB/GP neurons target A1, but also send a denser projection to secondary auditory areas. Thus, the effects of stimulating or inhibiting BF PV neurons surely extend beyond A1, and our cortical recordings may encompass also changes in secondary auditory populations, in addition to primary auditory cortex that was the main target of our study.

Chavez C, Zaborszky L. 2017. Basal forebrain cholinergic-auditory cortical network: Primary versus nonprimary auditory cortical areas. *Cereb Cortex* **27**. doi:10.1093/cercor/bhw091

The Rat Brain in Stereotaxic Coordinates. 1982. , The Rat Brain in Stereotaxic Coordinates. doi:10.1016/c2009-0-63235-9

Supplementary Material 3: Targeting of pNB/GP for investigation of basal forebrain tonotopy

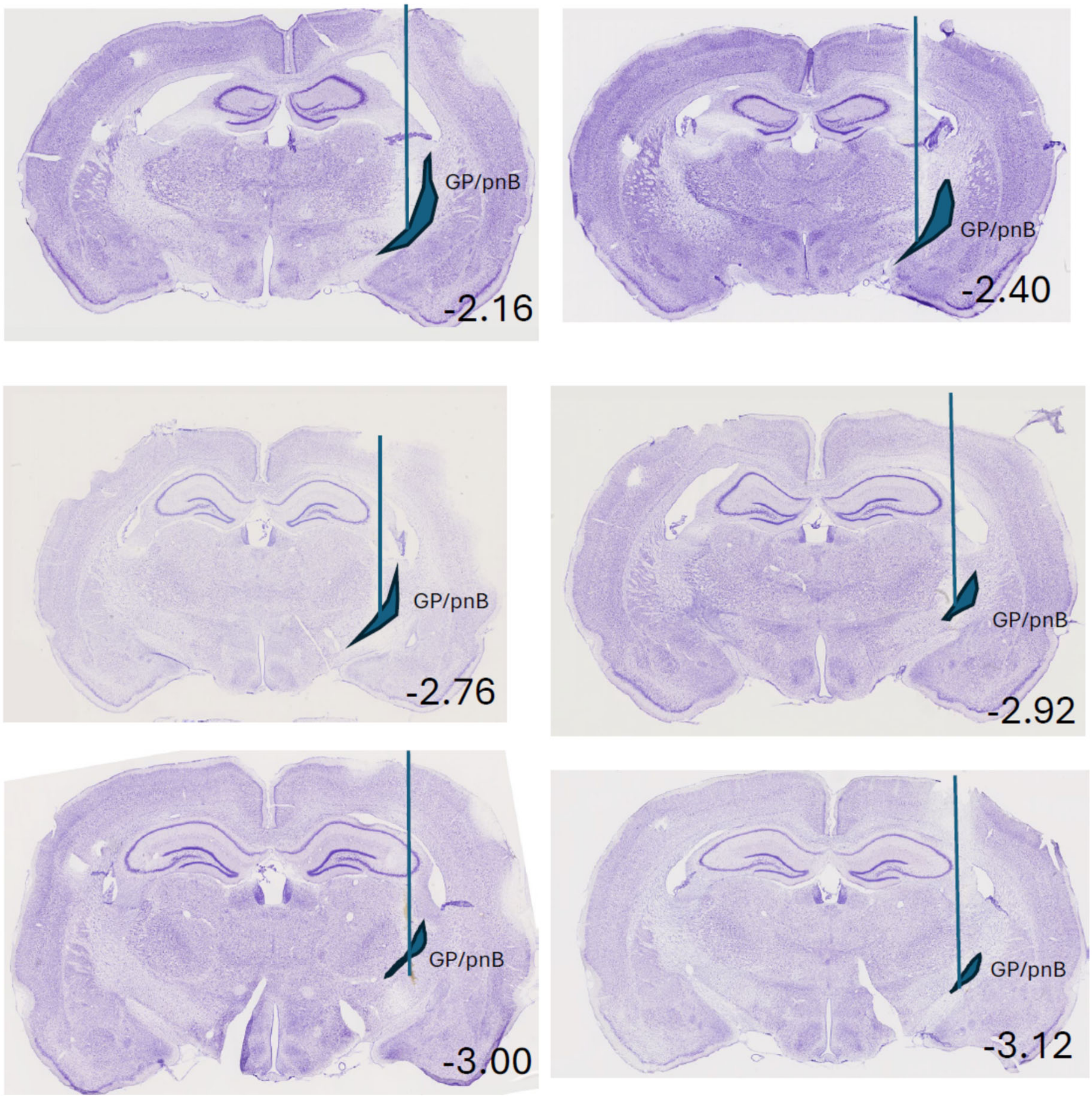

Examples of targeting during terminal electrophysiological studies of tonotopy in the basal forebrain at the indicated Bregma value according to the Paxinos-Watson atlas.

We illustrate here targeting along the anterior to posterior extent of the pNB/GP of the basal forebrain using electrode track reconstructions at representative AP values. Note that we typically made multiple penetrations in each animal; we show data here from six example animals out of a total of 18 animals from which we obtained data for basal forebrain tonotopy.

Supplementary Material 4: Quantification of mDlx Expression in subpopulations of pNB/GP neurons

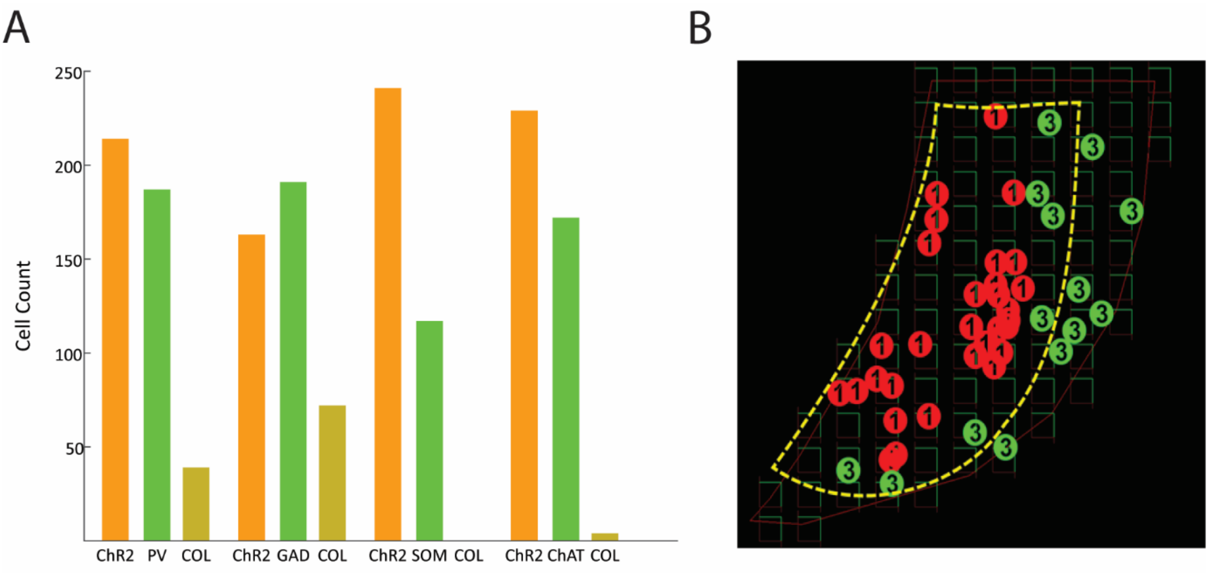

(A) counts of neurons expressing immunohistochemically identified parvalbumin (PV), GAD67 (GAD), or somatostatin (SOM), and their co-expression with the viral construct AAV5-mDLX-Chr2-mCherry, (ChR2). (B) Example BF section showing mDlx-labelled cells (red) and SOM+ cells (green). Yellow dashed line delineates GP border.

We performed immunohistochemistry and stereological counting in brain slices, see methods, at the location of the pNB/GP in two animals in order to determine the co-localization of the mDlx transfected neurons with PV+, GABA+, somatostatin+ and ChAT+ populations. Counts were performed separately for each cell type. For PV we counted 214 neurons that were positive for ChR2 and 187 neurons that were PV+. Of these 39 neurons were positive for both PV and ChR2, or 20% of the transfected neurons. For GAD67 we counted 163 neurons positive for ChR2, 191 positive for GAD67, and 84 expressed both markers, or 44% of the population. For somatostatin, 241 neurons were ChR2+, 117 were SOM+, with no neurons observed containing both markers. Finally for ChAT neurons we counted 229 ChR2+ neurons, 165 ChAT+ and 4 neurons expressed both markers, or 1.7%. Note that SOM+ neurons were largely absent from our region of interest pNB/GP, but were found nearby and more laterally near the GP border in the caudal part of the BF.

Supplementary Material 5: Local effects of optogenetic activation of BF PV neurons on frequency tuning within pNB/GP

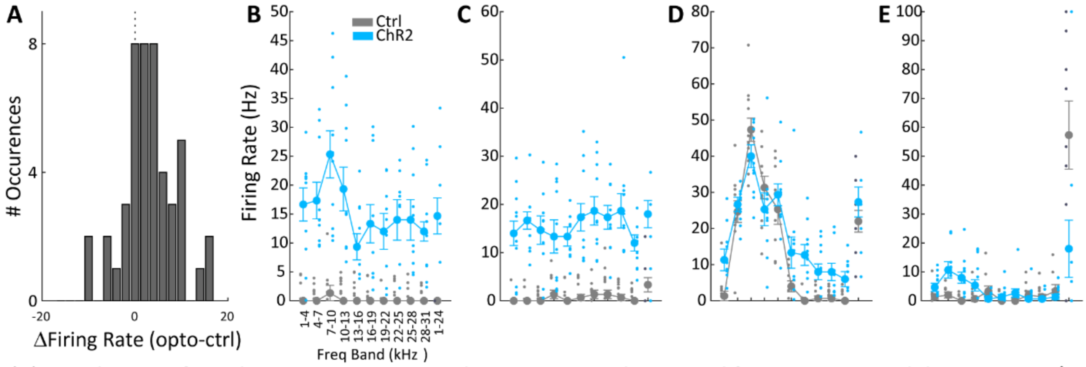

(A) Distribution of PV-ChR2 optogenetic stimulation vs. control on overal firing activity modulation in pNB/GP, illustrating the predominant excitation. Data is averaged across frequency bands. (B-C) Two example pNB/GP neurons illustrating general enhancement of activity during optogenetic activation. Small dots indicate individual data points (D-E) Two examples of pNB/GP neurons exhibiting an interaction between optogenetic activation and frequency tuning. Error bars SEM.

To assess the effect of ChR2 optogenetic activation on the activity of frequency-tuned neurons in pNB/GP, we performed a 2-way ANOVA with factors opto on/off (2 levels) and frequency band of auditory stimulation (11 levels). Out of a total of 171 neurons recorded in pNB/GP, we found that 33 exhibited a significant effect of light stimulation but no interaction (2-way ANOVA, $p<.05$ and $p>.1$), while 14 additional neurons exhibited a significant interaction between light stimulation and frequency (2-way ANOVA, $p<.05$). Taking these 47 neurons together, we observed an overall significant enhancement of activity following PV ChR2 activation compared to control conditions (paired t-test, $p<.001$, see Supp.Fig.5A). Two example neurons with a main effect of light stimulation are shown in Supp.Fig.5B-C, illustrating a general upward shift of neural activity triggered by laser activation, that seems to preserve frequency preference during control conditions. A minority of pNB/GP neurons showed more complex interactions between auditory and optogenetic activation, such as the neuron shown in Supp.Fig.5D, where optogenetic activation leads to activity enhancement only in frequency bands away from the characteristic frequency. The example activity in Supp.Fig.5E illustrates in addition suppression of activity during broad band auditory stimulation. The findings highlight that optogenetic activation tends to broadly upregulate neural activity in pNB/GP neurons. Diverse interactions between optogenetic and auditory activation are seen in a few cells, consistent with more complex recurrent activity dynamics within pNB/GP and synaptically coupled networks.

Supplementary Material 6: Viral Expression in PV+ axons

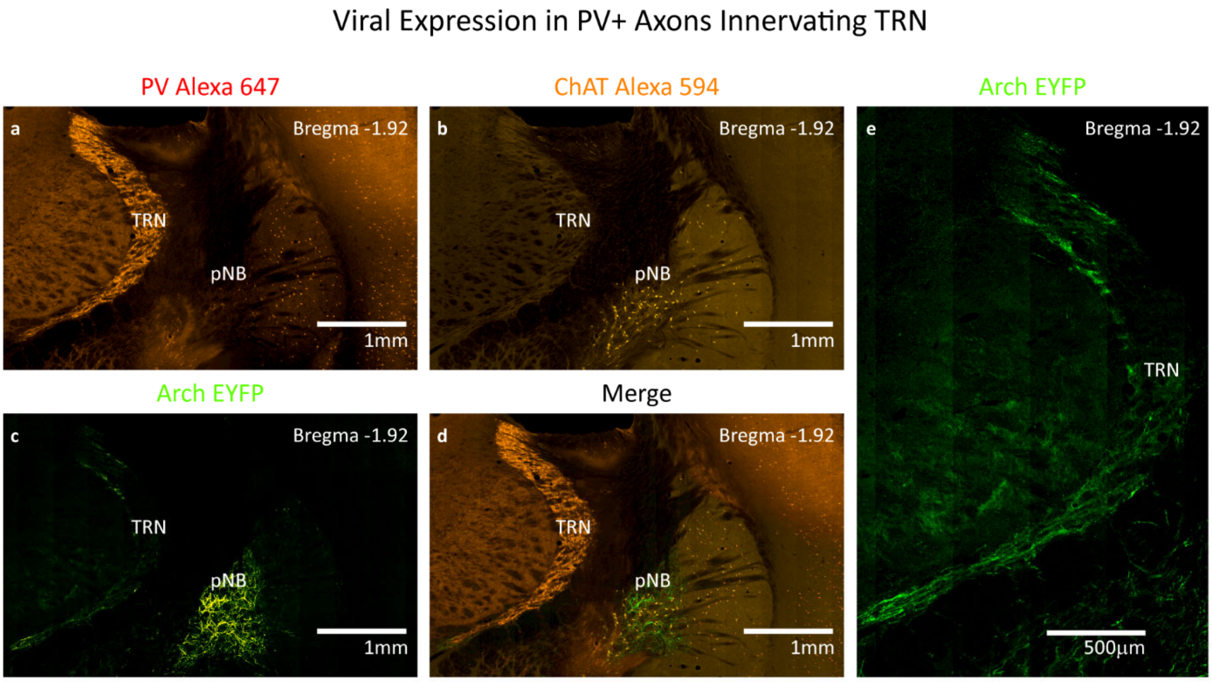

Immunostained section at the location of the pNB including the thalamic reticular nucleus (TRN). (a)Immunostaining for parvalbumin (PV), showing clearly the location of the TRN. (b) Immunostaining for choline acetyl transferase (ChAT), revealing a cluster of cholinergic cells at the putative site of the pNB. (c) Epifluorescent image showing the location within pNB of the ARCH viral construct containing the flurophore EYFP. (d) Merged image for the three flurophores. (e) Zoomed image of the TRN showing clear viral expression in axonal fibers.

Immunostaining for PV (a) clearly reveals the TRN, as well as showing PV+ neurons spread throughout the GP as expcted. Staining for ChAT (b) revealed clusters of cholinergic cells around the injection site that were non overlapping with the PV population seen in (a), see also Figure 1 B for quantification. The viral expression was mostly confined to pNB, with some spread into the surrounding GP as also evidenced by the location of the cholinergic cells in (b). The merged image in (d) shows the viral expression in relation to PV+ and ChAT+ neurons in the pNB. Axonal processes expressing EYFP can be seen in the zoomed image of the TRN in (e), indicating that pNB PV+ neurons are innervating the TRN.

We have no clear evidence of labelled fibers in the auditory cortex. The lack of expression in cortical terminal fields may be due to the tropism of the virus in these fibers or a failure to transfect BF PV corticopetal neurons. We believe that the former is a likely possibility, as there is robust viral expression in PV+ neurons and we could activate optotagged neurons using light stimulation at the viral transfection site.

Supplementary Material 7: MGB and AC responses to narrow band frequency target sounds with and without broadband mask

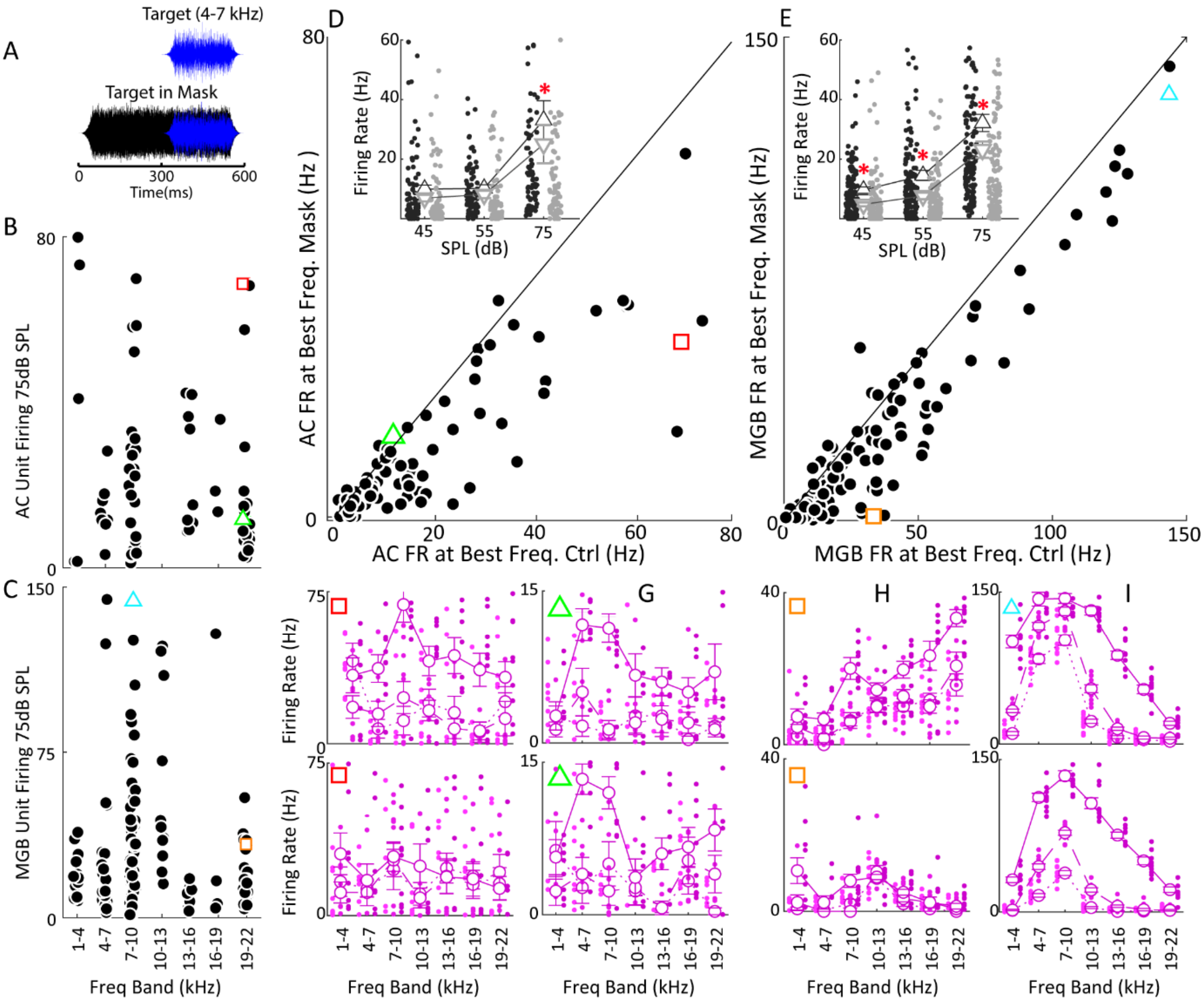

Effects of broadband noise masking on the responses of AC and MGB neurons to a narrow band noise signal. **(A)** Stimulation paradigm with and without mask. **(B)** Firing rates for all frequency tuned AC neurons in response their preferred frequency band at 100% amplitude. **(C)** Same as (B) but for MGB. **(D)** Firing rates of frequency-tuned AC neurons to their preferred frequency band versus responses to the same band in the presence of broadband mask. Insets show the mean response rates for different amplitudes, with (black) and without (magenta) broadband masking noise. **(E)** Same as D but for the MGB. **(F-G)** Mean firing rates across frequencies, and for 3 amplitudes (dotted: 20%, dashed 40%, solid 100%), of two example AC neurons illustrating degrees of neural response degradation in presence of the mask. Top: without mask (magenta), bottom: with mask (black), **(H-I)** Same as F, but for two MGB neurons. Colored symbols denote the example neurons in the different panels. Error bars SEM.

We obtained spiking activity from 145 and 79 neurons in MGB and AC respectively that were frequency selective in the control condition (2-way ANOVA with factors frequency and amplitude, main effect of frequency P<0.05). We observed that neurons in both areas generally tended to respond both to the mask and to the target sound. Examining the neural response to the preferred frequency at high amplitude in the control condition, we found that across both areas all the target sound frequency bands were represented (see Supp.Fig.7B,C). For MGB and AC at our electrode penetration sites, many neurons showed preference for low frequencies (7-10 kHz and lower bands). Based on these observations, we selected target sounds in the 4-10 kHz range for the further electrophysiological validation and used this frequency range and the corresponding implantation

coordinates for the subsequent behavioural studies. We proceeded to study the effect of the broadband mask on neural responses to the target sounds. The presence of the broadband mask strongly attenuated responses at the preferred frequency in AC and MGB (paired t-test, $p<0.01$, see Supp.Fig.7D,E, respectively). On average, the broadband mask tended to suppress activity for all three amplitudes in MGB, whereas suppression was most pronounced at high amplitudes in AC (Supp.Fig.7D,E, insets). The impact of the mask was variable across neurons, ranging from essentially abolishing target sound responses (Supp.Fig.7F,H) to having little impact (Supp.Fig.7G,I). Neural responses in both areas varied with target sound amplitude (one-way repeated-measures ANOVA, $P<0.05$, Supp.Fig.7D,E insets). Even at the lowest amplitude tested, significant auditory responses were apparent, particularly in the MGB. Therefore, we considered that 45dB amplitude level was close to perceptual threshold for this stimulus set.

Supplementary Material 8: Behavioral performance in PV-Arch group for 4-7Hz target sounds

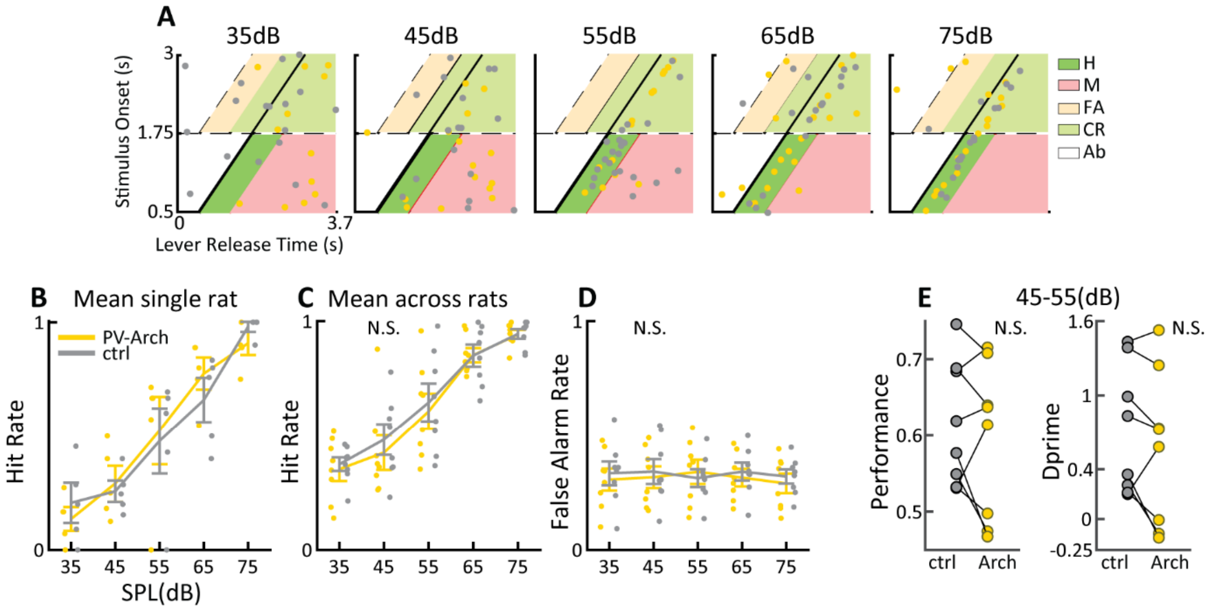

Supplementary Figure 8 *Behavioral effects of BF PV-Arch inhibition with 4-7khz targets.* **(A)** Lever release times for an example session at five different SPLs. Target onset is indicated by the thick, oblique line. The colored trapezoids refer to hit (H), miss (M), false alarm (FA) and correct rejection (CR) and abort (AB) responses (see legend). **(B)** Mean hit rate across all behavioral sessions (n=5) for the animal in (A) in the light stimulation vs. control condition. **(C,D)** Mean hit rate and false alarm rate across animals (n=8) using aggregate data from each rat . **(E)** Mean performance and d' for the same data using SPL near perceptual threshold. Error bars reflect SEM.

Supplementary Material 9: Behavioral performance in PV-ChR2 group for 4-7Hz target sounds

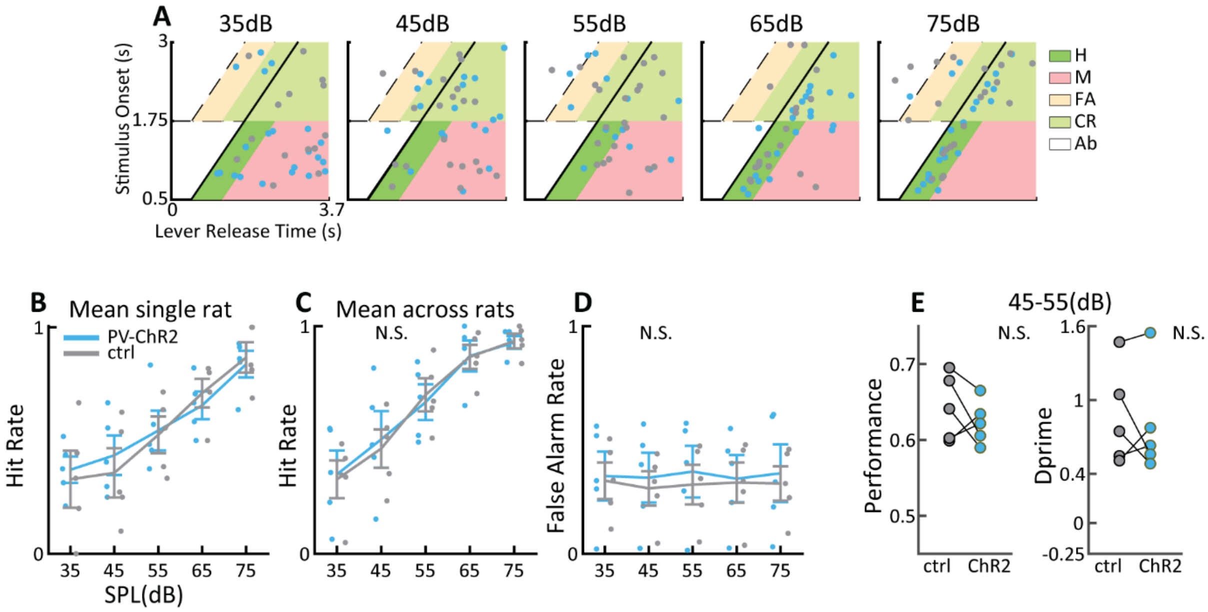

Supplementary Figure 9 *Behavioral effects of BF PV-ChR2 activation with 4-7khz targets.* **(A)** Lever release times for an example session at five different SPLs. Target onset is indicated by the thick, oblique line. The colored trapezoids refer to hit (H), miss (M), false alarm (FA) and correct rejection (CR) and abort (AB) responses (see legend). **(B)** Mean hit rate across all behavioral sessions (n=5) for the animal in (A) in the light stimulation vs. control condition. **(C,D)** Mean hit rate and false alarm rate across animals (n=5) using aggregate data from each rat. **(E)** Mean performance and d’ for the same data using SPL near perceptual threshold. Error bars reflect SEM.

Supplementary Material 10: Behavioral performance in mDlx-ChR2 group for 4-7Hz target sounds

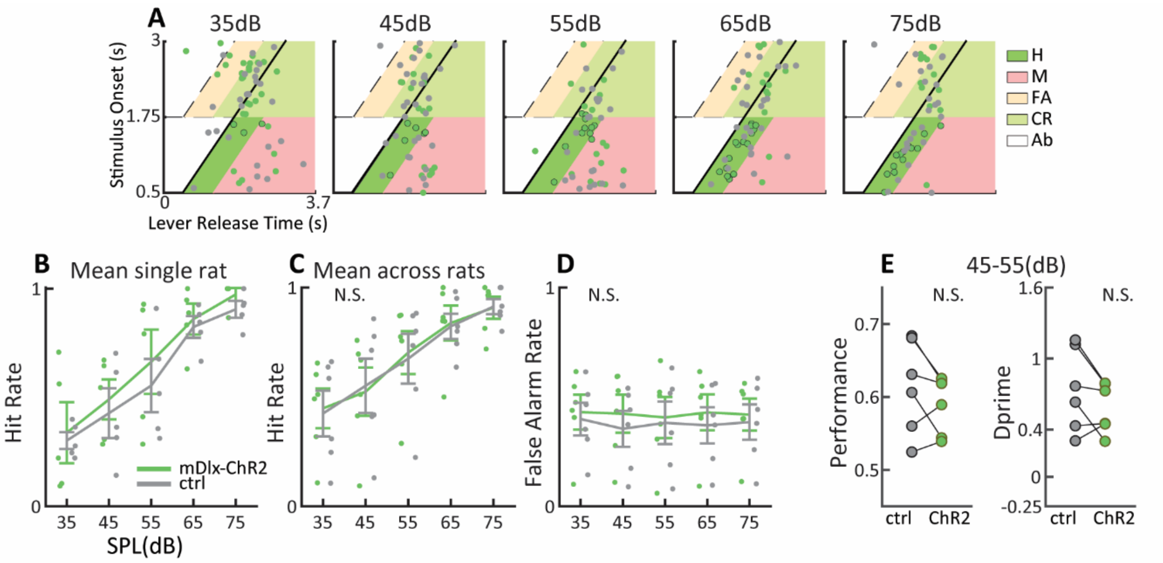

Supplementary Figure 10 *Behavioral effects of BF mDlx ChR2 activation with 4-7khz targets.* **(A)** Lever release times for an example session at five different SPLs. Target onset is indicated by the thick, oblique line. The colored trapezoids refer to hit (H), miss (M), false alarm (FA) and correct rejection (CR) and abort (AB) responses (see legend). **(B)** Mean hit rate across all behavioral sessions (n=5) for the animal in (A) in the light stimulation vs. control condition. **(C,D)** mean hit rate and false alarm rate across animals (n=6) using aggregate data from each rat. **(E)** Mean performance and d' for the same data using SPL near perceptual threshold. Error bars reflect SEM.

Supplementary Material 11: Histological localization of LED positions in the pNB/GP

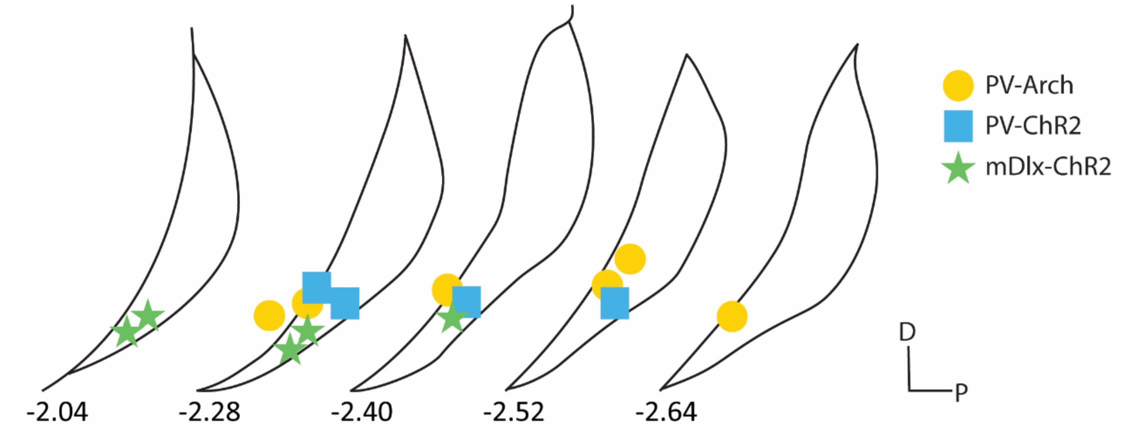

Supplementary Figure 11. *Location of LED placement in pNB/GP* . LED placement was determined from reconstructions of Nissl stained sections for all animals used in the behavioral experiments with differing viral conditions. PV neuronal inhibition using Archaerhopsin, PV neuronal excitation using ChR2, and general GABAergic neuronal excitation using mDlx. Numbers refer to AP coordinates in mm, the outline delineates the pNB/GP border at that AP location.

As we found behavioral effects exclusively in the PV-Arch group, we wanted to ensure that this was not due to a systematic difference in LED probe locations between the groups. We therefore reconstructed the LED locations from Nissl-stained sections for all animals, with the results shown in Supp. Fig. 11. This illustrates that LED locations were located within, or in close proximity above, the pNB/GP, clustering around -2.28mm AP and spanning an AP range of around 600μm. A one-way ANOVA [$F(2)=3.63$, $p>0.05$] showed no significant difference in the AP location of the LEDs between groups. This suggests that our behavioral findings are due to specific modulation of pNB/GP neuronal cell types, and do not result from any biases in LED probe placement. The LED probe locations tend to cluster in the ventral and anterior aspect of the pNB/GP, corresponding to the representation of higher auditory frequencies (compare Fig.3C). This may explain why behavioral effects were observed in the 7-10kHz but not the lower 4-7kHz frequency band.

Supplementary Table 1

Parameters for detection task training phase 2.

Step	Minimal Onset Time (ms)	Maximum Onset Time (ms)	Response window (ms)
1	150	450	1500
2	300	600	1000
3	400	100	900
4	500	1500	800
5	500	2000	800
6	500	3000	700
